# Supplementary material for: Efficient Single-Strand Break Repair Requires Binding to Both Poly(ADP-Ribose) and DNA by the Central BRCT Domain of XRCC1
Source: Cell Rep. 2019 Jan 15;26(3):573–581.e5. doi: 10.1016/j.celrep.2018.12.082 (PMC6334254; doi:10.1016/j.celrep.2018.12.082)
Supplement: Document S1. Figures S1–S4 [file mmc1.pdf]

Cell Reports, Volume *26*

Supplemental Information

Efficient Single-Strand Break Repair Requires Binding to Both Poly(ADP-Ribose) and DNA by the Central BRCT Domain of XRCC1

Luis M. Polo, Yingqi Xu, Peter Hornyak, Fernando Garces, Zhihong Zeng, Richard Hailstone, Steve J. Matthews, Keith W. Caldecott, Antony W. Oliver, and Laurence H. Pearl

FIGURE S1 - OLIGONUCLEOTIDE STRUCTURES AND AFFINITIES - related to FIGURE 1

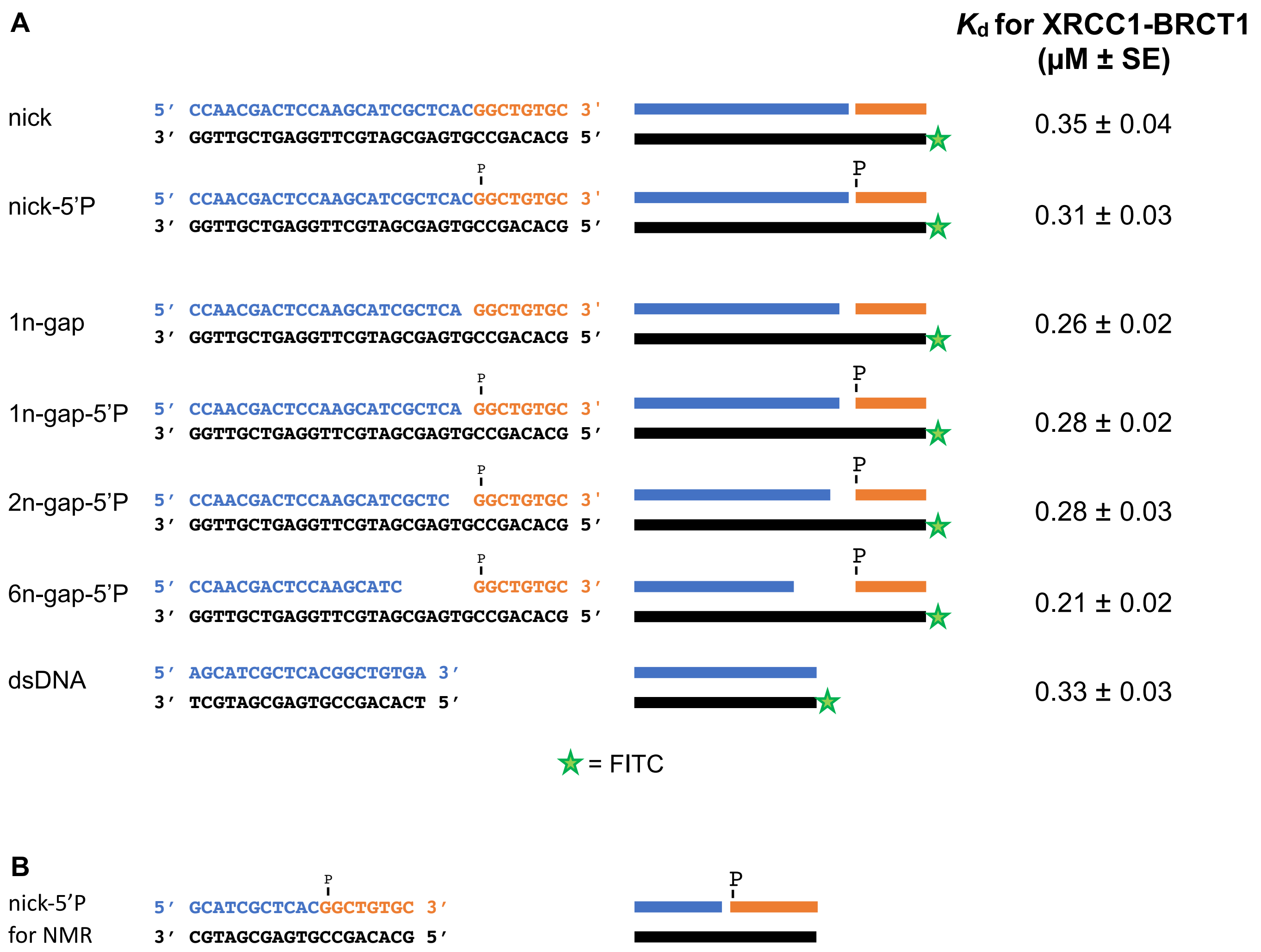

A. Schematics of DNA oligonucleotide duplexes used in this study, and their Kd for binding to XRCC1-BRCT1. The positions of 5'-phosphorylations are indicated by 'P' and the green star indicates the addition of the fluorescent dye fluorescein isothiocyanate (FITC).

B. Schematic of DNA oligonucleotide duplex used in NMR studies.

FIGURE S2 - Chemical Shift Perturbations - related to FIGURE 2

BRCT1
+ PAR4

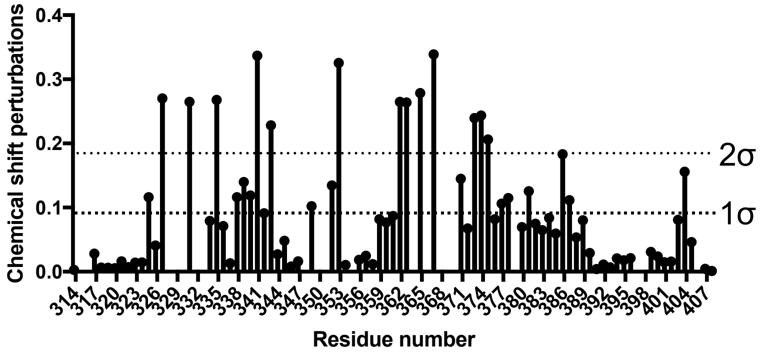

BRCT1
+ DNA

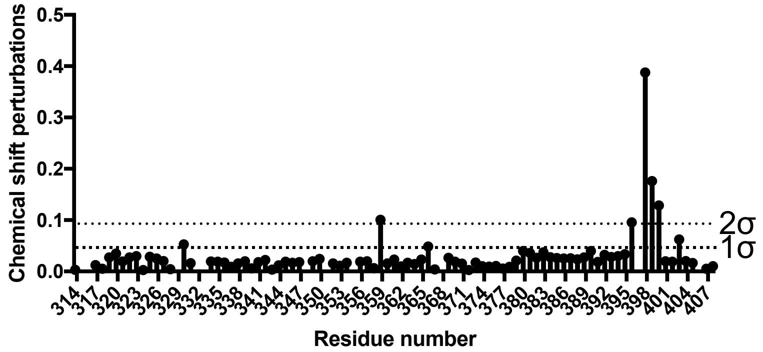

BRCT1-PAR4
+ DNA

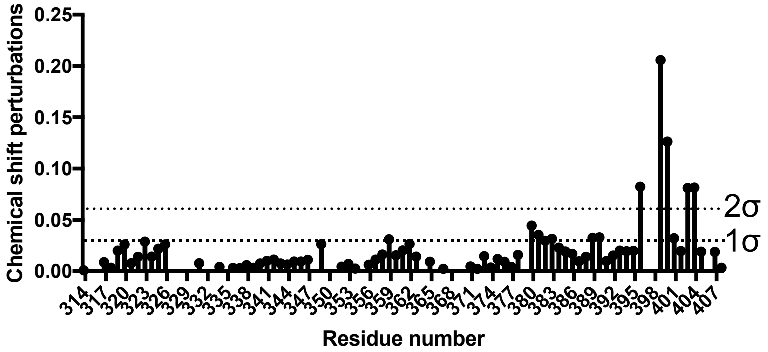

Chemical shift perturbations (CSP) by residue - residues whose CSP exceeds 2 standard deviations from the mean CSP for the experiment are highlighted in Figure 2e,f

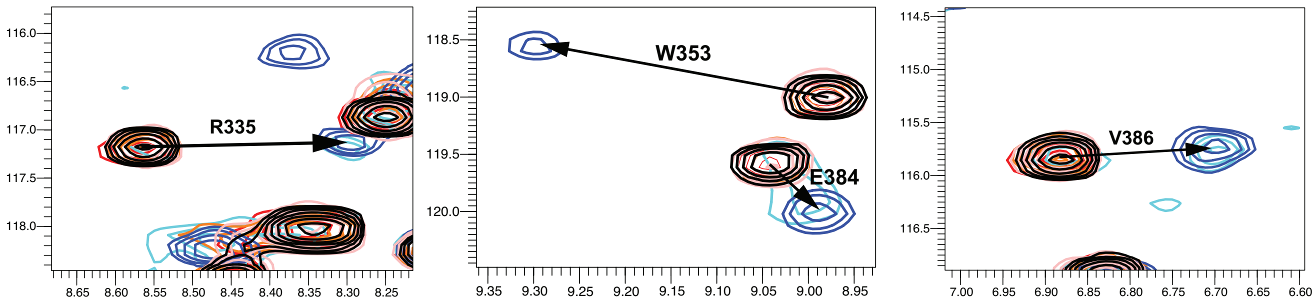

Protein:PAR	apo	1:1	1:2	1:4	1:10
Colour	black	pink	orange	cyan	blue

Example chemical shift perturbations (CSP) as a function of protein:PAR4 ratio. All shifts are fully saturated at 1:10 ratio.

FIGURE S3 - OLIGONUCLEOTIDE-BINDING AFFINITIES OF XRCC1-BRCT1 MUTANTS - related to FIGURE 3

	K_d for XRCC1-BRCT1 (μM ± SE)	
	oligonucleotide	
**XRCC1-BRCT1 Variant/Mutant**	**nick**	**nick 5'Pi**
R399	0.31 ± 0.04	0.32 ± 0.04
Q399	0.42 ± 0.1	1.45 ± 0.26
R399D/R400Q	not determined	not determined
R335A/K369A	0.33 ± 0.09	1.16 ± 0.32

Binding affinities of oligonucleotides (see FIGURE S1A) for different XRCC1-BRCT1 mutants/variants.

FIGURE S4 - XRCC1 EXPRESSION LEVELS AND COMET ASSAYS - related to FIGURE 4

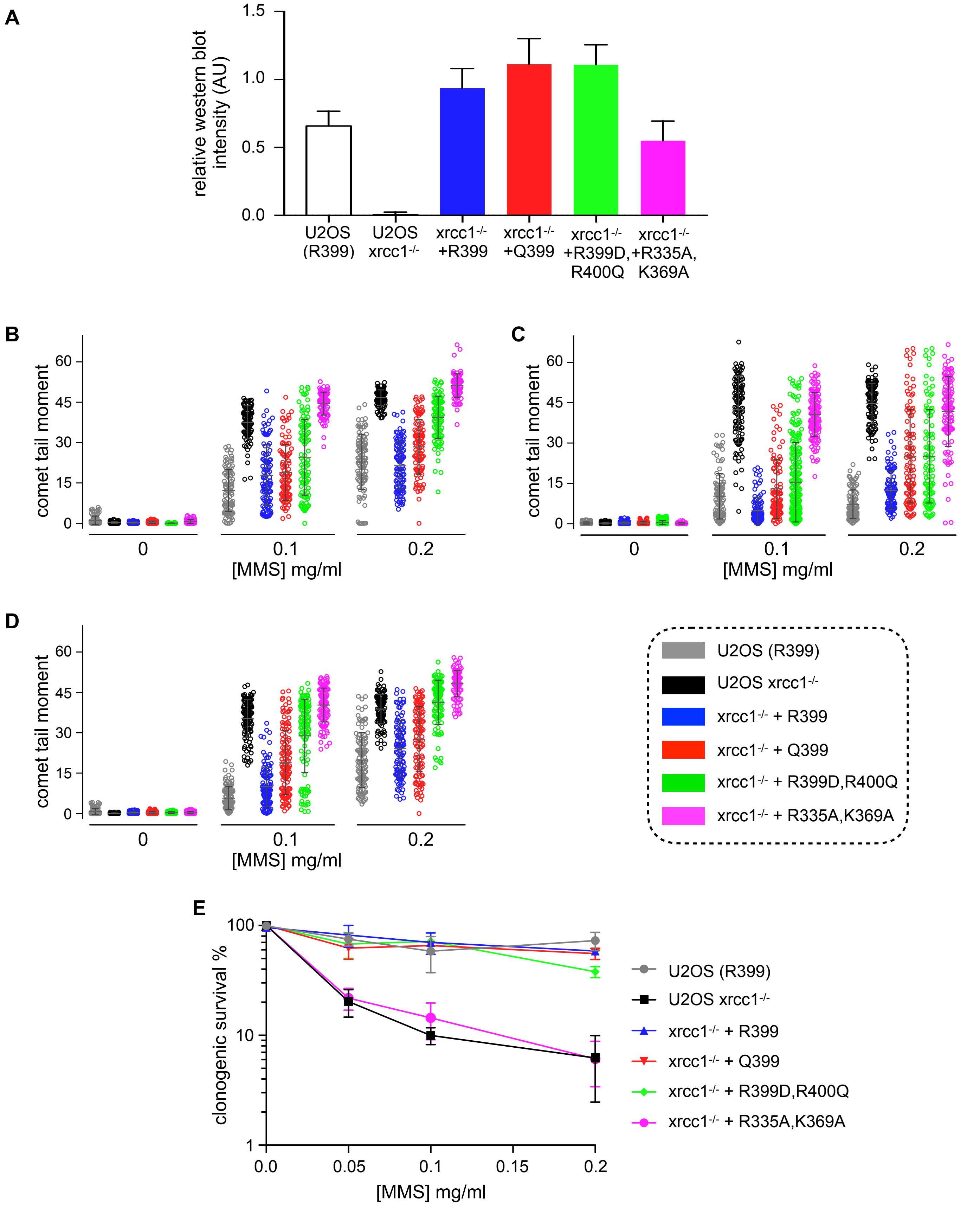

A. Relative expression levels of XRCC1 proteins determined by western blot intensity, in : U2OS cells, *XRCC1* gene edited cells, and gene edited cells with different XRCC1 variants or mutants added back. All mutants and variants are expressed at least at levels comparable to the wild-type U2OS cells.

B,C,D Replicate alkaline comet assays summarised in **FIGURE 4C.** The legend is shown in the dashed cartouche.

E. Clonogenic survival assay for U2OS variants and mutants (see **METHODS**).
