# Supplementary material for: Efficient Single-Strand Break Repair Requires Binding to Both Poly(ADP-Ribose) and DNA by the Central BRCT Domain of XRCC1
Source: Cell Rep. 2019 Jan 15;26(3):573–581.e5. doi: 10.1016/j.celrep.2018.12.082 (PMC6334254; doi:10.1016/j.celrep.2018.12.082)
Supplement: Document S2. Article plus Supplemental Information [file mmc2.pdf]

Cell Reports

Report

Efficient Single-Strand Break Repair Requires Binding to Both Poly(ADP-Ribose) and DNA by the Central BRCT Domain of XRCC1

Graphical Abstract

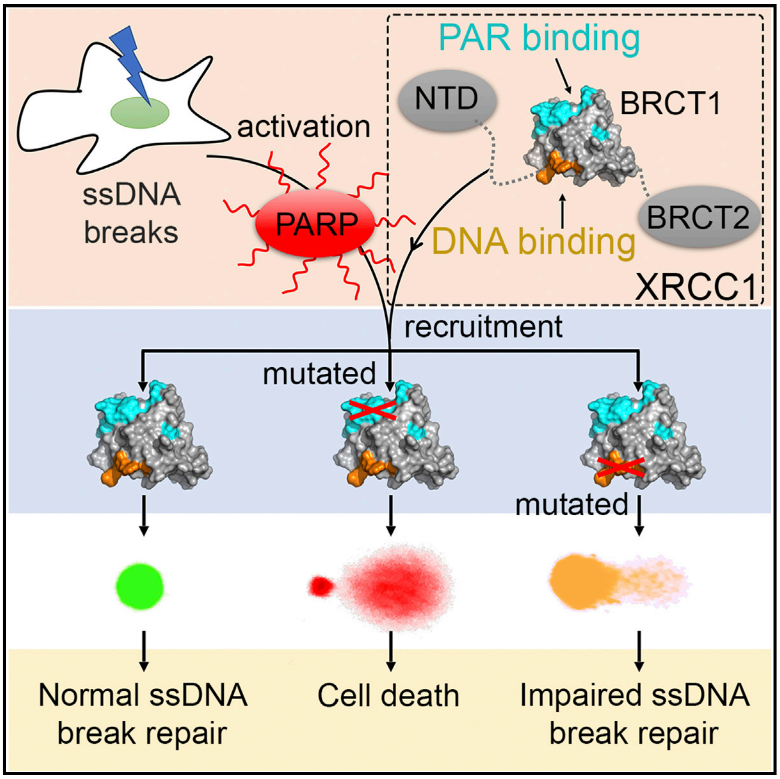

Highlights

- Interaction of XRCC1 with PAR and DNA are both mediated by the central BRCT domain
- Interaction with PAR and DNA occurs through non-overlapping binding surfaces
- Mutational disruption of DNA binding to XRCC1 impairs recruitment to DNA damage
- Disruption of DNA binding by XRCC1 impairs repair of DNA single-strand breaks

Authors

Luis M. Polo, Yingqi Xu, Peter Hornyak, ..., Keith W. Caldecott, Antony W. Oliver, Laurence H. Pearl

Correspondence

k.w.caldecott@sussex.ac.uk (K.W.C.), antony.oliver@sussex.ac.uk (A.W.O.), laurence.pearl@sussex.ac.uk (L.H.P.)

In Brief

Polo et al. map the binding sites for poly(ADP-ribose) and DNA of XRCC1 to non-overlapping surfaces of the central BRCT domain. Mutational disruption of the DNA binding site, which contains a common human polymorphism, affects recruitment and retention of XRCC1 at DNA damage and impairs single-strand break repair *in vivo*.

Polo et al., 2019, Cell Reports *26*, 573–581
January 15, 2019 © 2018 The Authors.
https://doi.org/10.1016/j.celrep.2018.12.082

CellPress

OPEN ACCESS
CellPress

Efficient Single-Strand Break Repair Requires Binding to Both Poly(ADP-Ribose) and DNA by the Central BRCT Domain of XRCC1

Luis M. Polo,[1] Yingqi Xu,[2] Peter Hornyak,[1,3,5] Fernando Garces,[1,6] Zhihong Zeng,[3] Richard Hailstone,[3] Steve J. Matthews,[2] Keith W. Caldecott,[3,*] Antony W. Oliver,[1,*] and Laurence H. Pearl[1,4,7,*]
[1]Cancer Research UK DNA Repair Enzymes Group, Genome Damage and Stability Centre, School of Life Sciences, University of Sussex, Falmer, Brighton BN1 9RQ, UK
[2]Cross-Faculty NMR Centre, Department of Life Sciences, Faculty of Natural Sciences, Imperial College London, London SW7 2AZ, UK
[3]Genome Damage and Stability Centre, School of Life Sciences, University of Sussex, Falmer, Brighton BN1 9RQ, UK
[4]Division of Structural Biology, Institute of Cancer Research, Chester Beatty Laboratories, 237 Fulham Road, London SW1E 6BT, UK
[5]Present address: Vascular Venture Kft., Molecular Biology Laboratories, Budapest 1022, Hungary
[6]Present address: Department of Therapeutics Discovery, Amgen Research, Amgen Inc., 1 Amgen Center Drive, Thousand Oaks, CA 91320, USA
[7]Lead Contact
*Correspondence: k.w.caldecott@sussex.ac.uk (K.W.C.), antony.oliver@sussex.ac.uk (A.W.O.), laurence.pearl@sussex.ac.uk (L.H.P.)
https://doi.org/10.1016/j.celrep.2018.12.082

SUMMARY

XRCC1 accelerates repair of DNA single-strand breaks by acting as a scaffold protein for the recruitment of Polβ, LigIIIα, and end-processing factors, such as PNKP and APTX. XRCC1 itself is recruited to DNA damage through interaction of its central BRCT domain with poly(ADP-ribose) chains generated by PARP1 or PARP2. XRCC1 is believed to interact directly with DNA at sites of damage, but the molecular basis for this interaction within XRCC1 remains unclear. We now show that the central BRCT domain simultaneously mediates interaction of XRCC1 with poly(ADP-ribose) and DNA, through separate and non-overlapping binding sites on opposite faces of the domain. Mutation of residues within the DNA binding site, which includes the site of a common disease-associated human polymorphism, affects DNA binding of this XRCC1 domain *in vitro* and impairs XRCC1 recruitment and retention at DNA damage and repair of single-strand breaks *in vivo*.

INTRODUCTION

X-ray repair cross-complementing protein 1 (XRCC1) is a scaffold protein that coordinates the repair of DNA single-strand nicks and gaps (single strand breaks [SSBs]; Caldecott, 2003). It constitutively associates with a DNA polymerase (Polβ) and a DNA ligase (Lig3α) to fill and ligate the broken strand (Caldecott et al., 1994, 1996; Kubota et al., 1996; Nash et al., 1997) and recruits the end-processing enzymes polynucleotide kinase-3′-phosphatase (PNKP) and aprataxin (APTX), which ensure the presence of 3′-hydroxyl and 5′-phosphate groups at gap margins (Ahel et al., 2006; Jilani et al., 1999; Loizou et al., 2004).

Recruitment of XRCC1 complexes to sites of DNA damage is strongly dependent on activation of the DNA-damage-responsive poly(ADP-ribose) polymerases PARP1 and PARP2 (El-Khamisy et al., 2003; Hanzlikova et al., 2017; Mortusewicz et al., 2007; Schreiber et al., 2002). PARP-dependent recruitment of XRCC1 requires the central BRCT domain (BRCT1), which conserves components of a pocket similar to the phosphopeptide-binding BRCT domains in proteins such as TOPBP1 (Rappas et al., 2011; Wardlaw et al., 2014). However, rather than interacting with phosphorylated proteins, the phosphate-binding pocket in XRCC1-BRCT1 has been shown to mediate interaction with the poly(ADP-ribose) (PAR) chains generated by PARP1 or PARP2 (Breslin et al., 2015; Li et al., 2013).

Although an interaction with PAR plays a major role in recruiting XRCC1 to sites of DNA damage, several studies have suggested that XRCC1 is able to interact directly with DNA (Mani et al., 2004; Nazarkina et al., 2007a, 2007b; Ström et al., 2011) and that this plays a role in its DNA repair function (Berquist et al., 2010; Wei et al., 2013). Previous NMR studies implicated the N-terminal domain of XRCC1 in high-affinity interactions with gapped DNA molecules (Marintchev et al., 1999), but subsequent work has cast doubt on this, and there is currently no coherent understanding of which part of XRCC1 is involved (London, 2015). We show here that both PAR and DNA interactions are mediated by non-overlapping binding sites on the first of the two BRCT domains in XRCC1 (BRCT1). Targeted mutations in the DNA-binding site, which contains a common human polymorphism, impair XRCC1 interaction with DNA *in vitro* and markedly affect the kinetics of XRCC1 recruitment, its retention on damaged chromatin, and the efficiency of DNA single-strand break repair *in vivo*. These data resolve a critical unanswered question in the field.

RESULTS

XRCC1 Binds DNA through BRCT1

Previous studies had suggested that the N-terminal domain (NTD) of XRCC1, which is required for association of Polβ with

This is an open access article under the CC BY license (http://creativecommons.org/licenses/by/4.0/).

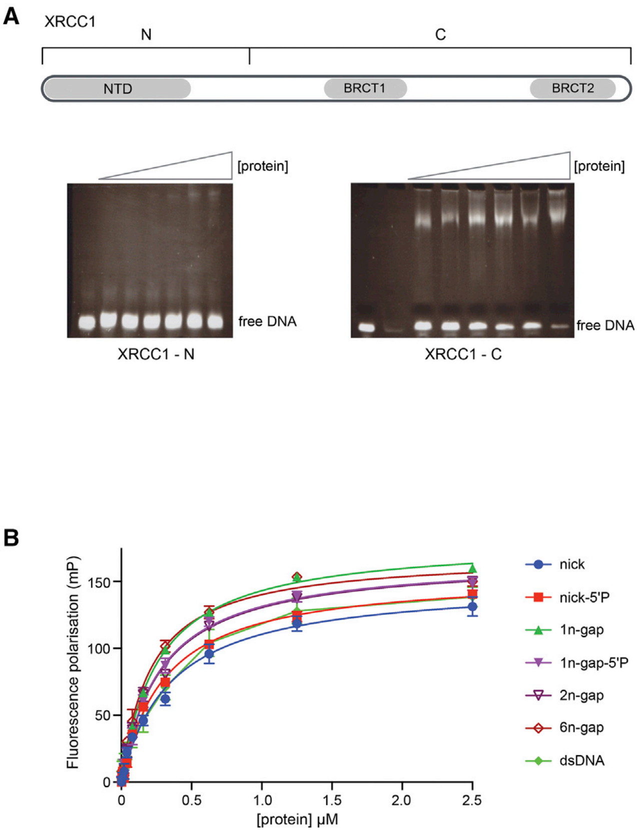

Figure 1. XRCC1-BRCT1 Binds DNA

(A) Electromobility shift assay (EMSA) shows that the ability to bind DNA resides in the C-terminal region of XRCC1 containing the two BRCT domains rather than the N-terminal region as previously suggested (Marintchev et al., 1999).

(B) Fluorescence polarization assay of XRCC1-BRCT1 binding to various fluorescein isothiocyanate (FITC)-labeled dsDNA oligonucleotides. No substantial differences in affinity were observed between intact, nicked, and gapped molecules, which all bound with sub-micromolar affinity. Oligonucleotide structures and K_d values for their binding to XRCC1-BRCT1 are shown in Figure S1A. Data represent the mean of four measurements comprised of two separate replicates with XRCC1-BRCT1 from two separate protein purifications. Error bars show ± 1 standard error of the mean (SEM).

XRCC1, possesses an inherent affinity for DNA with single-strand nicks and short gaps (Marintchev et al., 1999). To discover whether other parts of XRCC1 might also be involved, we expressed and purified separate N-terminal (residues 1–223) and C-terminal (224–631) constructs of murine XRCC1 and examined their ability to interact with a 39-base-pair DNA duplex containing a single-strand nick, in an electrophoretic mobility shift assay (EMSA) (see STAR Methods). Contrary to the published model, we were unable to detect any significant interaction in EMSAs with the construct containing the NTD domain. By contrast, the C-terminal construct lacking the putative DNA binding NTD produced robust EMSA band shifts (Figure 1A). The marked difference in behavior of the two parts of XRCC1 suggests that its inherent DNA-binding ability resides in the C-terminal region, which incorporates the two BRCT domains, rather than in the Polβ-binding N-terminal domain.

As BRCT domains in other proteins have been implicated in binding to DNA (Leung and Glover, 2011 and references therein), and as PAR and DNA have many structural and chemical features in common, we considered the notion that BRCT1, which mediates the interaction of XRCC1 with PAR (Breslin et al., 2015; Li et al., 2013), might also bind DNA. To address this, we expressed and purified the isolated BRCT1 domain of human XRCC1 and assessed its interaction with DNA using a fluorescence polarization assay (see STAR Methods). We observed robust interaction of XRCC1-BRCT1 with a blunt-ended double-stranded DNA (dsDNA) oligonucleotide and a variety of different "damaged" dsDNA molecules with K_d values in the range ~0.2–0.4 μM (Figures 1B and S1). Oligonucleotides incorporating single-strand gaps bound slightly more tightly than the nicked or intact oligonucleotides, but the presence or absence of 5′-phosphate groups at the nick or gap had little effect on the affinity of the interaction.

Mapping PAR- and DNA-Binding Sites on XRCC1-BRCT1

We previously showed that mutation of residues in XRCC1-BRCT1 that are topologically equivalent to phosphate-binding residues in other BRCT domains disrupted the interaction of XRCC1 with PAR (Breslin et al., 2015). To further characterize the PAR-binding site, we recorded two-dimensional (2D) ^{1}H–^{15}N heteronuclear single quantum coherence (HSQC) NMR spectra on isotopically labeled samples of human XRCC1-BRCT1 (see STAR Methods) and measured chemical shift perturbations in the presence of a purified PAR oligomer (PAR4) (see STAR Methods; Figures 2A, 2B, and S2). We observed significant chemical shift perturbations in residues within and proximal to the putative phosphate-binding pocket, including Arg 335 and Lys 369, whose mutation disrupts binding to PAR *in vitro* and XRCC1 recruitment to DNA damage *in vivo* (Breslin et al., 2015 and see below), confirming our identification of this pocket as critical for PAR binding. The exchange behavior of the chemical shift perturbations observed were in the slow-exchange range, suggesting an affinity for PAR4 in the sub-micromolar range, consistent with previous observations (Kim et al., 2015).

^{1}H–^{15}N HSQC spectra recorded in the presence of a nicked dsDNA oligonucleotide with the internal 5′ end phosphorylated (see STAR Methods) instead of PAR also display clear chemical shift changes consistent with the sub-micromolar affinity of the nicked DNA for XRCC1-BRCT1 observed in the fluorescence polarization experiments (see above) and confirming an interaction between XRCC1-BRCT1 and DNA. However, most of the observed perturbations upon DNA binding occurred in residues that were not strongly affected by PAR (Figure 2C), suggesting that the DNA and PAR molecules were binding to distinct sites on XRCC1-BRCT1. We tested this by titrating in increasing amounts of nicked dsDNA into XRCC1-BRCT1 already saturated by PAR4 and observed a pattern of chemical shift perturbations that represented the superposition of perturbations observed for the separate additions of PAR and DNA to protein alone (Figure 2D).

Mapped onto the NMR solution structure of XRCC1-BRCT1 (PDB: 2D8M), the sets of residues perturbed by binding of PAR or by binding of DNA define distinct non-overlapping patches on the solvent accessible surface of the domain (Figures 2E and 2F). The residues perturbed by PAR binding lie on the face

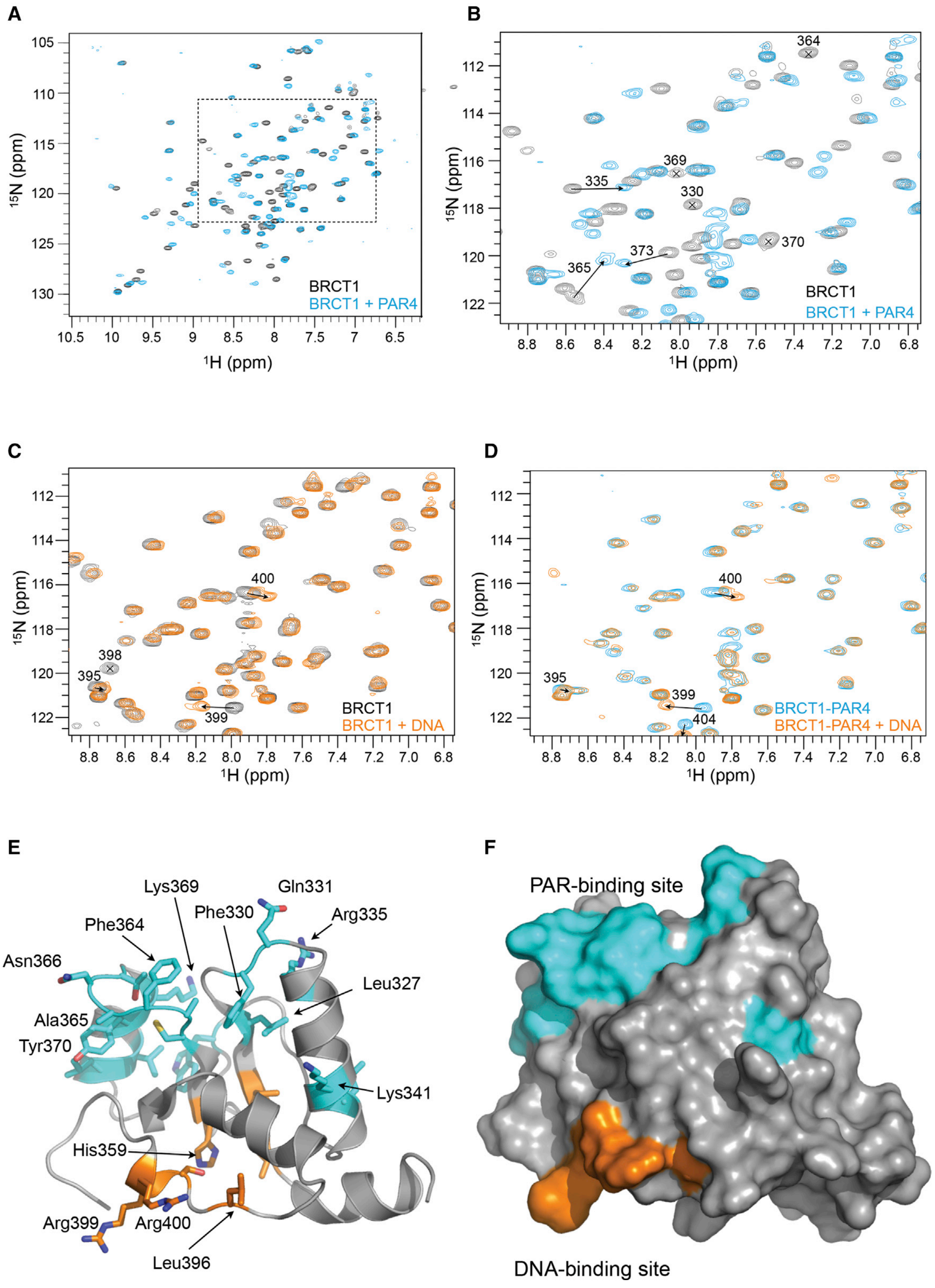

Figure 2. Mapping PAR- and DNA-Binding Sites

(A) ^{1}H–^{15}N heteronuclear single quantum coherence (HSQC) NMR spectra for XRCC1-BRCT1 alone (black) overlayed with the HSQC spectrum for XRCC1-BRCT1 in the presence of a fragment of poly(ADP-ribose)—PAR4 (cyan; see STAR Methods). Assignments for these and other spectra have been deposited in the Biological Magnetic Resonance Bank (BRMB: 27598).

(legend continued on next page)

of the domain formed by the C-terminal end of the central parallel β sheet and map in and around the phosphate-binding "pocket," which is conserved in many BRCT domains that mediate interaction with phosphorylated peptide motifs (Leung and Glover, 2011). The residues perturbed by DNA binding localize to the opposite face of the domain within the N-terminal ends of the β strands and from a segment of polypeptide extending from the C-terminal α helix.

Mutation Analysis of the DNA-Binding Site

Next, we sought to validate the results of the NMR experiments by exploring the effect of disruptive mutations in the predicted DNA-binding site on biochemical and functional assays. In the absence of a high-resolution structure for a complex, predicting a single point mutation that abrogates XRCC1-BRCT1 interaction with DNA, as we have been able to do with phosphopeptide interactions with other BRCT domains (Qu et al., 2013; Rappas et al., 2011), is challenging. However, the highly basic nature of the surface patch revealed by NMR titration experiments with DNA suggests that mutations altering the electrostatics should affect interaction of the XRCC1-BRCT1 domain with DNA (Figure 3A). We therefore mutated a number of residues in this region that were perturbed by DNA binding in the NMR studies and found that an XRCC1-BRCT1-R399D,R400Q double mutant, which would be expected to substantially disrupt the basic nature of the putative DNA-binding site without perturbing the structure of the domain, could be readily expressed and purified as a soluble protein.

Human populations have a common CAG → CGG polymorphism in codon 399 (allele frequency between 16%–35%), which results in a glutamine rather than an arginine in the expressed protein (Hu et al., 2005). Multiple studies have suggested association of the G/G and A/G genotypes with enhanced susceptibility to a broad range of cancer types (Casse et al., 2003; Divine et al., 2001; Mateuca et al., 2008; Mittal et al., 2008; Natukula et al., 2013) and/or variable responses to chemotherapy (Deng et al., 2015; Li and Li, 2013; Singh et al., 2017; Wu et al., 2012). However, other studies and meta-analyses have failed to demonstrate such association, and the significance of this common polymorphism remains controversial (Jacobs and Bracken, 2012; Taylor et al., 2002; Yuan et al., 2010; Zeng et al., 2013). Because the participation of this polymorphic residue in DNA binding provides the first suggestion of a biochemical role, we compared Gln399 and Arg399 variants of the XRCC1-BRCT1 for functionality, alongside the R399D/R400Q double mutant.

Using a previously described assay (Breslin et al., 2015), we tested the ability of the XRCC1-BRCT1 constructs to bind to PAR chains generated on histone H1 by PARP1 in the presence of NAD^+ (see STAR Methods; Figure 3B). PAR binding by the DNA-binding site double mutant and the Gln399 variant were essentially identical to that of the Arg399 XRCC1-BRCT1 domain, whereas a construct with a previously described double mutation in the PAR-binding pocket (R335A, K369A; Breslin et al., 2015) failed to interact with PAR. These data demonstrate that the DNA-binding site identified by the NMR titration experiments does not contribute significantly to the interaction with PAR and confirms that neither the double mutation nor the polymorphic variation have any substantial effect on the three-dimensional structure and consequent functional integrity of the BRCT domain.

By contrast, although both codon 399 variants and the PAR-binding pocket mutant protein displayed low or sub-micromolar affinity for 5′-phosphorylated or unphosphorylated nicked dsDNA in a fluorescence polarization assay (see STAR Methods), the R399D,R400Q double mutant failed to bind DNA, confirming the critical involvement of these residues in DNA binding by XRCC1-BRCT1 (Figures 3C and S3).

DNA Binding Is Required for XRCC1-Dependent Repair

To determine whether the ability of XRCC1-BRCT1 to bind DNA plays a role in its function as a DNA repair scaffold, we employed U2OS cells in which the *XRCC1* gene was disrupted by CRISPR/Cas9-mediated gene editing and XRCC1 expression then restored in the edited cells by transfection with wild-type or mutant EGFP-XRCC1 fusion protein (see STAR Methods).

We observed robust and rapid recruitment of both R399 and Q399 variants of the EGFP-XRCC1 fusion to DNA damage caused by laser micro-irradiation in these cell lines (see STAR Methods), whereas we failed to detect recruitment of the PAR-binding-defective R335A,K369A double mutant, as previously described (Breslin et al., 2015). The R399D,R400Q double mutant that is competent for PAR binding but defective in DNA binding (see above) was still recruited to DNA damage. However, this occurred with markedly slower kinetics than the native variants (Figure 4A). Chromatin retention of the EGFP-XRCC1 fusion protein following DNA damage was also strongly affected by mutational disruption of the DNA-binding site in BRCT1, with the R399D,R400Q double mutant being as poorly retained as the PAR-binding defective R335A,K369A mutant (Figure 4B).

Finally, we looked at the ability of the variant and mutant XRCC1 proteins to support DNA repair in U2OS cells following

(B) Close up of boxed region in (A), highlighting residues in and around the putative phosphate-binding pocket in XRCC1-BRCT1, whose chemical shift changes on binding of PAR4.

(C) Close up of equivalent region to (B), showing the HSQC spectra for XRCC1-BRCT1 alone (black), overlayed with the HSQC spectrum for XRCC1-BRCT1 in the presence of a 19-mer dsDNA with a 5′-phosphorylated nick on one strand, 8 nucleotides in from the 3′ end (orange)—see Figure S1. Residues whose chemical shifts change on binding of the dsDNA are highlighted.

(D) As (C) but showing the overlay of HSQC spectra for XRCC1-BRCT1 bound to PAR4 (cyan) with that of XRCC1-BRCT1 + PAR4 with the addition of nicked, 5′-phosphorylated dsDNA (orange). Residues that display a change in chemical shift on binding of dsDNA to XRCC1-BRCT1 alone display very similar shifts when the dsDNA is added to XRCC1-BRCT1 already bound to PAR4, showing that the binding sites for PAR4 and dsDNA are non-overlapping and that these two ligands are not mutually competitive.

(E) Secondary structure cartoon of the NMR structure of XRCC1-BRCT1 (PDB: 2D8M), with residues showing perturbed peptide backbone chemical shifts on PAR4 binding highlighted in cyan and those whose chemical shifts are perturbed by binding of nicked dsDNA, highlighted in orange. Highlighted residues are those whose chemical shift perturbation exceeds 2 SD of the average chemical shift across the whole domain or those where the peak becomes broadened.

(F) As (E) but with a solvent-accessible surface representation showing the non-overlapping binding sites for PAR and for dsDNA on opposite faces of the domain.

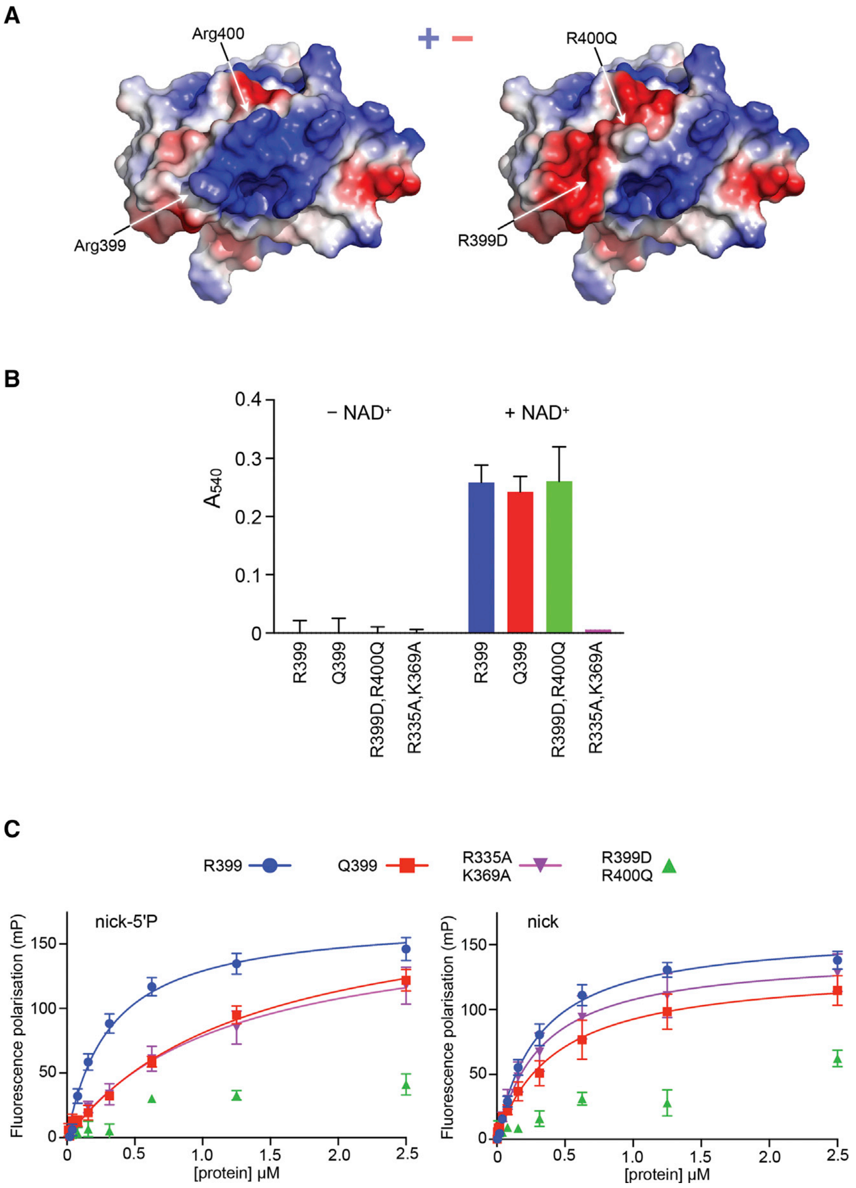

Figure 3. Mutational Analysis of the DNA-Binding Site

(A) Solvent-accessible surface of the DNA-binding site colored by electrostatic potential (calculated in PyMol). Residues perturbed by DNA binding (including Arg399 and Arg400) map to an intensely positively charged surface patch (left), whose polarity is predicted to be reversed by the combination of R399D and R400Q mutations (right).

(B) PAR-binding assay (see STAR Methods) of XRCC1-BRCT1 variants and mutants. Both codon 399 variants and the putative DNA binding disruptive R399D,R400Q double mutant bind tightly to PAR chains generated on plates coated with histone H1 and incubated with PARP1 and NAD^+, whereas no binding is seen with the R335A,K369A double mutant, which affects two residues in the PAR-binding site (Breslin et al., 2015). No binding is seen for any of the constructs in the absence of NAD^+. Data represent the mean of four measurements of three separate replicates analyzed by two-way ANOVA. Error bars show ± 1 SEM.

(C) Fluorescence polarization assays of XRCC1-BRCT1 variants and mutants to FITC-labeled nicked dsDNA oligonucleotides with (left) or without (right) 5′ phosphorylation at the nick site. The codon 399 variants and the PAR-binding site mutant all bind with high affinity to both nicked duplex oligonucleotides, whereas the R399D,R400Q double mutant shows very low fluorescence poloarization (FP) values, which cannot be fitted to a binding curve (for K_d values, see Figure S1B). Data represent the mean of four measurements comprised of two separate replicates with XRCC1-BRCT1 from two separate protein purifications. Error bars show ± 1 SEM.

treatment with varying doses of methyl methanesulfonate (MMS), using an alkaline comet assay that reports on unrepaired DNA SSBs (Breslin et al., 2006). Wild-type U2OS cells (which contain the R399 *XRCC1* variant) in which the endogenous *XRCC1* gene was disrupted by gene editing accumulated far higher levels of SSBs than did wild-type U2OS cells (Figures 4C and S4B–S4D). The SSB repair defect in these *XRCC1* gene-edited cells was effectively rescued by expression of either of the residue 399 polymorphic variants of EGFP-XRCC1, but not by the PAR-binding defective R335A,K369A double mutant (Figure 4C). Expression of the PAR-binding competent but DNA-binding-defective R399D,R400Q mutant resulted in an intermediate level of SSB repair that was significantly reduced compared to wild-type U2OS cells.

DISCUSSION

A direct consequence of the activation of PARP1 and/or PARP2 at DNA strand breaks is the rapid formation of PAR chains covalently anchored primarily to the PARP enzymes themselves (Caldecott, 2008; Daniels et al., 2015). A primary function of these PAR chains in the context of DNA repair is the recruitment of the XRCC1 scaffold protein to sites of DNA damage (London, 2015; Li et al., 2013; Breslin et al., 2015; Hanzlikova et al., 2017). XRCC1-dependent repair of single-strand DNA breaks generated by oxidative damage, alkylation, or abortive topoisomerase 1 activity requires the catalytic activity of up to four associated DNA repair enzymes (Polβ, Lig3α, PNKP, and APTX), each of which requires access to the 5′ and/or 3′ termini at the margins of the DNA break to perform its particular reaction. To facilitate this, XRCC1 functions as a DNA-binding scaffold protein to help recruit, retain, and coordinate its partner enzymes at the site of damage once PARP1 or PARP2 are released.

The results we present here unambiguously identify the central BRCT domain as both necessary and sufficient for DNA binding by XRCC1 and resolve a long-standing question in the field. The

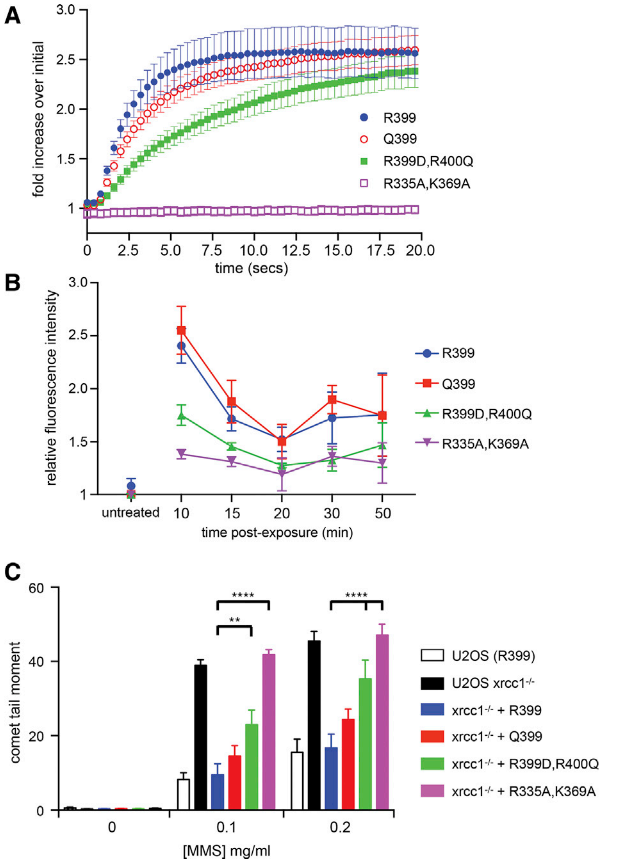

Figure 4. DNA Binding Contributes to XRCC1-Dependent DNA Damage Repair

(A) Recruitment of XRCC1 variants and mutants to laser micro-irradiation DNA damage. Both codon 399 variants are rapidly recruited to sites of DNA damage in U2OS cells transiently transfected with GFP-XRCC1 and accumulate to comparable levels over 15–20 s post-laser exposure. Consistent with previous studies (Breslin et al., 2015), mutational disruption of XRCC1 PAR binding (R335A,K369A) abolishes XRCC1 recruitment to DNA damage in this time frame. The R399D,R400Q mutant, which is fully competent for PAR binding but defective for DNA binding *in vitro*, still accumulates at sites of damage but with markedly slower kinetics than the DNA-binding and PAR-binding competent constructs. Error bars are SEM for 30 cells analyzed for each curve, except for the R399D,R400Q mutant, where only 10 cells were analyzed.

(B) Retention of XRCC1 at DNA damage. Both codon 399 variants showed high levels of retention on chromatin in U2OS cells stably transfected with GFP-XRCC1 10–20 min after exposure to DNA damage by hydrogen peroxide, whereas the PAR-binding defective mutant shows much lower levels. The DNA-binding defective mutant is retained at higher levels than the PAR-binding defective mutant but markedly reduced in comparison to the unmutated variants. Data represent the mean of three measurements, with >8000 cells per sample per experiment using Perkin-Elmer Operetta software and analysed by two-way ANOVA. Error bars show ± 1 SEM.

(C) Untransformed U2OS cells, which carry the R399 XRCC1 variant, display moderate dose-dependent alkaline comet tail moments (see STAR Methods) after treatment with methyl methanesulfonate (MMS), indicative of unrepaired single-strand breaks (SSBs). U2OS cells where the XRCC1 gene is disrupted by CRISPR/Cas9 gene editing and consequently expresses undetectable levels of XRCC1 protein (Figure S2) show significantly larger tail moments indicative of much higher levels of SSBs. This repair defect can be substantially rescued by expression of GFP-XRCC1 with either codon 399 variant, but not by GFP-XRCC1 with the PAR-binding defect. Consistent with its much reduced DNA binding *in vitro*, its slower recruitment to laser damage, and its poorer chromatin retention post-damage, the R399D,R400Q mutant is significantly less able to rescue SSB repair in the xrcc1$^{-/-}$ cells. Error bars indicate SEM over three replicates (Figure S3). Average tail moments from 100 cells/sample were measured using Comet Assay IV software (Perceptive Instruments, UK) and were scored blind. Data are the average of three independent experiments. Error bars show ± 1 SEM.

DNA-binding site in the BRCT domain is distinct from the binding site for PAR, which interacts with the conserved pocket that mediates phosphopeptide binding in BRCT domain proteins, such as BRCA1, 53BP1, and TOPBP1 (Baldock et al., 2015; Clapperton et al., 2004; Kilkenny et al., 2008; Leung et al., 2011; Qu et al., 2013; Shiozaki et al., 2004; Sun et al., 2017; Williams et al., 2004) and DNA end binding in RFC1 (Kobayashi et al., 2006). Furthermore, the PAR-binding and DNA-binding sites on BRCT1 are non-overlapping, so that both polymers can interact with XRCC1 simultaneously. This would allow a smooth transfer from PAR to DNA as the main anchor for retaining XRCC1 at the site of damage, while its partner enzymes process and repair the DNA break. Consistent with this model, we find that DNA binding, although not essential for recruitment of XRCC1 downstream of PARP activation, contributes to XRCC1 recruitment and retention on damaged chromatin. *In vivo*, this is reflected in a significant reduction in the efficiency of SSB repair. However, like some other XRCC1 mutations that affect SSB repair efficiency (Breslin and Caldecott, 2009; Loizou et al., 2004), disruption of DNA binding does not significantly impact cell survival (Figure S4E), probably due to the ability of homologous recombination to compensate for reduced SSB repair during S phase (Caldecott, 2008).

The DNA-binding site we have identified on XRCC1-BRCT1 encompasses residue 399, which has a common Arg/Gln genetic polymorphism in human populations. The significance of this polymorphism is a matter of considerable study and debate, but there is no clear consensus as to whether or not the less common Q399 variant predisposes individuals to a variety of cancers or whether it predicts a better response to a variety of genotoxic chemotherapies—both of which are claimed in the literature. Our data do show small differences in DNA binding and damage recruitment between the Q399 and R399 variants of XRCC1, with the Q399 variant being overall less effective in SSB repair than the R399 variant (Figure 4C), but none of these differences achieve statistical significance in our hands. Nonetheless, the involvement of this polymorphic residue in a defined biochemical function of XRCC1 may provide a more mechanistic basis for assessing its importance.

Our results reinforce the role of XRCC1 as a spatial organizer of SSB repair, providing a stable protein scaffold on DNA in the vicinity of a break that is completely independent of the highly specific and competing interactions of its partner enzymes with the 3′ and 5′ termini at the margins of the break. How this competition is structurally orchestrated and coordinated by XRCC1 to achieve efficient SSB repair remains to be determined.

STAR★METHODS

Detailed methods are provided in the online version of this paper and include the following:

- KEY RESOURCES TABLE
- CONTACT FOR REAGENT AND RESOURCE SHARING
- EXPERIMENTAL MODEL AND SUBJECT DETAILS
  - Cell culture
- METHOD DETAILS
  - Cloning
  - Expression and purification
  - Poly(ADP-ribose) preparation
  - NMR resonance assignment
  - Electrophoretic Mobility Shift Assay
  - Fluorescence polarization
  - Poly (ADP-ribose) binding assays
  - UVA-laser micro-irradiation
  - Generation of gene-edited U2-OS cells
  - Cell lines expressing XRCC1
  - Alkaline comet assays
  - Chromatin associated XRCC1
  - Cell survival assay
  - SDS-PAGE and western blotting
- QUANTIFICATION AND STATISTICAL ANALYSIS
- DATA AND SOFTWARE AVAILABILITY

SUPPLEMENTAL INFORMATION

Supplemental Information includes four figures and can be found with this article online at https://doi.org/10.1016/j.celrep.2018.12.082.

ACKNOWLEDGMENTS

This work was supported by Cancer Research UK Programme grants C302/A14532 and C302/A24386 (A.W.O. and L.H.P.), MRC (MR/J006750/1) and CR-UK (A16771) Programme grants (K.W.C.), and Wellcome Trust Senior Investigator Award (100280) and multiuser equipment grant (104833; S.J.M.).

AUTHOR CONTRIBUTIONS

Conceptualization, L.M.P., L.H.P., A.W.O., and K.W.C.; Methodology, L.H.P., A.W.O., K.W.C., S.J.M., and L.M.P.; Investigation, L.M.P., Y.X., P.H., F.G., Z.Z., R.H., and A.W.O.; Writing – Original Draft, L.H.P.; Writing – Review & Editing, L.H.P., A.W.O., K.W.C., S.J.M., and L.M.P.; Visualization, L.M.P., A.W.O., and L.H.P.; Supervision, L.H.P., A.W.O., K.W.C., and S.J.M.; Funding Acquisition, L.H.P., A.W.O., K.W.C., and S.J.M.

DECLARATION OF INTERESTS

The authors declare no competing interests.

Received: October 1, 2018
Revised: November 26, 2018
Accepted: December 18, 2018
Published: January 15, 2019

REFERENCES

Ahel, I., Rass, U., El-Khamisy, S.F., Katyal, S., Clements, P.M., McKinnon, P.J., Caldecott, K.W., and West, S.C. (2006). The neurodegenerative disease protein aprataxin resolves abortive DNA ligation intermediates. Nature *443*, 713–716.

Baldock, R.A., Day, M., Wilkinson, O.J., Cloney, R., Jeggo, P.A., Oliver, A.W., Watts, F.Z., and Pearl, L.H. (2015). ATM localization and heterochromatin repair depend on direct interaction of the 53BP1-BRCT2 domain with γH2AX. Cell Rep. *13*, 2081–2089.

Berquist, B.R., Singh, D.K., Fan, J., Kim, D., Gillenwater, E., Kulkarni, A., Bohr, V.A., Ackerman, E.J., Tomkinson, A.E., and Wilson, D.M., 3rd. (2010). Functional capacity of XRCC1 protein variants identified in DNA repair-deficient Chinese hamster ovary cell lines and the human population. Nucleic Acids Res. *38*, 5023–5035.

Breslin, C., and Caldecott, K.W. (2009). DNA 3′-phosphatase activity is critical for rapid global rates of single-strand break repair following oxidative stress. Mol. Cell. Biol. *29*, 4653–4662.

Breslin, C., Clements, P.M., El-Khamisy, S.F., Petermann, E., Iles, N., and Caldecott, K.W. (2006). Measurement of chromosomal DNA single-strand breaks and replication fork progression rates. Methods Enzymol. *409*, 410–425.

Breslin, C., Hornyak, P., Ridley, A., Rulten, S.L., Hanzlikova, H., Oliver, A.W., and Caldecott, K.W. (2015). The XRCC1 phosphate-binding pocket binds poly (ADP-ribose) and is required for XRCC1 function. Nucleic Acids Res. *43*, 6934–6944.

Caldecott, K.W. (2003). XRCC1 and DNA strand break repair. DNA Repair (Amst.) *2*, 955–969.

Caldecott, K.W. (2008). Single-strand break repair and genetic disease. Nat. Rev. Genet. *9*, 619–631.

Caldecott, K.W., McKeown, C.K., Tucker, J.D., Ljungquist, S., and Thompson, L.H. (1994). An interaction between the mammalian DNA repair protein XRCC1 and DNA ligase III. Mol. Cell. Biol. *14*, 68–76.

Caldecott, K.W., Aoufouchi, S., Johnson, P., and Shall, S. (1996). XRCC1 polypeptide interacts with DNA polymerase beta and possibly poly (ADP-ribose) polymerase, and DNA ligase III is a novel molecular ‘nick-sensor’ in vitro. Nucleic Acids Res. *24*, 4387–4394.

Casse, C., Hu, Y.C., and Ahrendt, S.A. (2003). The XRCC1 codon 399 Gln allele is associated with adenine to guanine p53 mutations in non-small cell lung cancer. Mutat. Res. *528*, 19–27.

Clapperton, J.A., Manke, I.A., Lowery, D.M., Ho, T., Haire, L.F., Yaffe, M.B., and Smerdon, S.J. (2004). Structure and mechanism of BRCA1 BRCT domain recognition of phosphorylated BACH1 with implications for cancer. Nat. Struct. Mol. Biol. *11*, 512–518.

Daniels, C.M., Ong, S.E., and Leung, A.K. (2015). The promise of proteomics for the study of ADP-ribosylation. Mol. Cell *58*, 911–924.

Deng, J.H., Deng, J., Shi, D.H., Ouyang, X.N., and Niu, P.G. (2015). Clinical outcome of cisplatin-based chemotherapy is associated with the polymorphisms of GSTP1 and XRCC1 in advanced non-small cell lung cancer patients. Clin. Transl. Oncol. *17*, 720–726.

Divine, K.K., Gilliland, F.D., Crowell, R.E., Stidley, C.A., Bocklage, T.J., Cook, D.L., and Belinsky, S.A. (2001). The XRCC1 399 glutamine allele is a risk factor for adenocarcinoma of the lung. Mutat. Res. *461*, 273–278.

El-Khamisy, S.F., Masutani, M., Suzuki, H., and Caldecott, K.W. (2003). A requirement for PARP-1 for the assembly or stability of XRCC1 nuclear foci at sites of oxidative DNA damage. Nucleic Acids Res. *31*, 5526–5533.

Elliott, R.J.R., Jarvis, A., Rajasekaran, M.B., Menon, M., Bowers, L., Boffey, R., Bayford, M., Firth-Clark, S., Key, R., Aqil, R., et al. (2015). Design and discovery of 3-aryl-5-substituted-isoquinolin-1-ones as potent tankyrase inhibitors. MedChemComm *6*, 1687–1692.

Hanzlikova, H., Gittens, W., Krejcikova, K., Zeng, Z., and Caldecott, K.W. (2017). Overlapping roles for PARP1 and PARP2 in the recruitment of endogenous XRCC1 and PNKP into oxidized chromatin. Nucleic Acids Res. *45*, 2546–2557.

Hu, Z., Ma, H., Chen, F., Wei, Q., and Shen, H. (2005). XRCC1 polymorphisms and cancer risk: a meta-analysis of 38 case-control studies. Cancer Epidemiol. Biomarkers Prev. *14*, 1810–1818.

Jacobs, D.I., and Bracken, M.B. (2012). Association between XRCC1 polymorphism 399 G->A and glioma among Caucasians: a systematic review and meta-analysis. BMC Med. Genet. *13*, 97.

Jilani, A., Ramotar, D., Slack, C., Ong, C., Yang, X.M., Scherer, S.W., and Lasko, D.D. (1999). Molecular cloning of the human gene, PNKP, encoding a polynucleotide kinase 3′-phosphatase and evidence for its role in repair of DNA strand breaks caused by oxidative damage. J. Biol. Chem. *274*, 24176–24186.

Kilkenny, M.L., Doré, A.S., Roe, S.M., Nestoras, K., Ho, J.C.Y., Watts, F.Z., and Pearl, L.H. (2008). Structural and functional analysis of the Crb2-BRCT2 domain reveals distinct roles in checkpoint signaling and DNA damage repair. Genes Dev. *22*, 2034–2047.

Kim, I.K., Stegeman, R.A., Brosey, C.A., and Ellenberger, T. (2015). A quantitative assay reveals ligand specificity of the DNA scaffold repair protein XRCC1 and efficient disassembly of complexes of XRCC1 and the poly(ADP-ribose) polymerase 1 by poly(ADP-ribose) glycohydrolase. J. Biol. Chem. *290*, 3775–3783.

Kobayashi, M., Figaroa, F., Meeuwenoord, N., Jansen, L.E., and Siegal, G. (2006). Characterization of the DNA binding and structural properties of the BRCT region of human replication factor C p140 subunit. J. Biol. Chem. *281*, 4308–4317.

Kubota, Y., Nash, R.A., Klungland, A., Schär, P., Barnes, D.E., and Lindahl, T. (1996). Reconstitution of DNA base excision-repair with purified human proteins: interaction between DNA polymerase beta and the XRCC1 protein. EMBO J. *15*, 6662–6670.

Leung, C.C., and Glover, J.N. (2011). BRCT domains: easy as one, two, three. Cell Cycle *10*, 2461–2470.

Leung, C.C., Gong, Z., Chen, J., and Glover, J.N. (2011). Molecular basis of BACH1/FANCJ recognition by TopBP1 in DNA replication checkpoint control. J. Biol. Chem. *286*, 4292–4301.

Li, K., and Li, W. (2013). Association between polymorphisms of XRCC1 and ADPRT genes and ovarian cancer survival with platinum-based chemotherapy in Chinese population. Mol. Cell. Biochem. *372*, 27–33.

Li, M., Lu, L.Y., Yang, C.Y., Wang, S., and Yu, X. (2013). The FHA and BRCT domains recognize ADP-ribosylation during DNA damage response. Genes Dev. *27*, 1752–1768.

Loizou, J.I., El-Khamisy, S.F., Zlatanou, A., Moore, D.J., Chan, D.W., Qin, J., Sarno, S., Meggio, F., Pinna, L.A., and Caldecott, K.W. (2004). The protein kinase CK2 facilitates repair of chromosomal DNA single-strand breaks. Cell *117*, 17–28.

London, R.E. (2015). The structural basis of XRCC1-mediated DNA repair. DNA Repair (Amst.) *30*, 90–103.

Mani, R.S., Karimi-Busheri, F., Fanta, M., Caldecott, K.W., Cass, C.E., and Weinfeld, M. (2004). Biophysical characterization of human XRCC1 and its binding to damaged and undamaged DNA. Biochemistry *43*, 16505–16514.

Marintchev, A., Mullen, M.A., Maciejewski, M.W., Pan, B., Gryk, M.R., and Mullen, G.P. (1999). Solution structure of the single-strand break repair protein XRCC1 N-terminal domain. Nat. Struct. Biol. *6*, 884–893.

Mateuca, R.A., Roelants, M., Iarmarcovai, G., Aka, P.V., Godderis, L., Tremp, A., Bonassi, S., Fenech, M., Bergé-Lefranc, J.L., and Kirsch-Volders, M. (2008). hOGG1(326), XRCC1(399) and XRCC3(241) polymorphisms influence micronucleus frequencies in human lymphocytes in vivo. Mutagenesis *23*, 35–41.

Mittal, R.D., Singh, R., Manchanda, P.K., Ahirwar, D., Gangwar, R., Kesarwani, P., and Mandhani, A. (2008). XRCC1 codon 399 mutant allele: a risk factor for recurrence of urothelial bladder carcinoma in patients on BCG immunotherapy. Cancer Biol. Ther. *7*, 645–650.

Mortusewicz, O., Amé, J.-C., Schreiber, V., and Leonhardt, H. (2007). Feedback-regulated poly(ADP-ribosyl)ation by PARP-1 is required for rapid response to DNA damage in living cells. Nucleic Acids Res. *35*, 7665–7675.

Nash, R.A., Caldecott, K.W., Barnes, D.E., and Lindahl, T. (1997). XRCC1 protein interacts with one of two distinct forms of DNA ligase III. Biochemistry *36*, 5207–5211.

Natukula, K., Jamil, K., Pingali, U.R., Attili, V.S., and Madireddy, U.R. (2013). The codon 399 Arg/Gln XRCC1 polymorphism is associated with lung cancer in Indians. Asian Pac. J. Cancer Prev. *14*, 5275–5279.

Nazarkina, ZhK., Khodyreva, S.N., Marsin, S., Radicella, J.P., and Lavrik, O.I. (2007a). Study of interaction of XRCC1 with DNA and proteins of base excision repair by photoaffinity labeling technique. Biochemistry (Mosc.) *72*, 878–886.

Nazarkina, Z.K., Khodyreva, S.N., Marsin, S., Lavrik, O.I., and Radicella, J.P. (2007b). XRCC1 interactions with base excision repair DNA intermediates. DNA Repair (Amst.) *6*, 254–264.

Qu, M., Rappas, M., Wardlaw, C.P., Garcia, V., Ren, J.Y., Day, M., Carr, A.M., Oliver, A.W., Du, L.L., and Pearl, L.H. (2013). Phosphorylation-dependent assembly and coordination of the DNA damage checkpoint apparatus by Rad4(TopBP1). Mol. Cell *51*, 723–736.

Rappas, M., Oliver, A.W., and Pearl, L.H. (2011). Structure and function of the Rad9-binding region of the DNA-damage checkpoint adaptor TopBP1. Nucleic Acids Res. *39*, 313–324.

Schreiber, V., Amé, J.C., Dollé, P., Schultz, I., Rinaldi, B., Fraulob, V., Ménissier-de Murcia, J., and de Murcia, G. (2002). Poly(ADP-ribose) polymerase-2 (PARP-2) is required for efficient base excision DNA repair in association with PARP-1 and XRCC1. J. Biol. Chem. *277*, 23028–23036.

Shiozaki, E.N., Gu, L., Yan, N., and Shi, Y. (2004). Structure of the BRCT repeats of BRCA1 bound to a BACH1 phosphopeptide: implications for signaling. Mol. Cell *14*, 405–412.

Singh, A., Singh, N., Behera, D., and Sharma, S. (2017). Polymorphism in XRCC1 gene modulates survival and clinical outcomes of advanced North Indian lung cancer patients treated with platinum-based doublet chemotherapy. Med. Oncol. *34*, 64.

Skinner, S.P., Fogh, R.H., Boucher, W., Ragan, T.J., Mureddu, L.G., and Vuister, G.W. (2016). CcpNmr AnalysisAssign: a flexible platform for integrated NMR analysis. J. Biomol. NMR *66*, 111–124.

Ström, C.E., Mortusewicz, O., Finch, D., Parsons, J.L., Lagerqvist, A., Johansson, F., Schultz, N., Erixon, K., Dianov, G.L., and Helleday, T. (2011). CK2 phosphorylation of XRCC1 facilitates dissociation from DNA and single-strand break formation during base excision repair. DNA Repair (Amst.) *10*, 961–969.

Sun, L., Huang, Y., Edwards, R.A., Yang, S., Blackford, A.N., Niedzwiedz, W., and Glover, J.N.M. (2017). Structural insight into BLM recognition by TopBP1. Structure *25*, 1582–1588.e3.

Tan, E.S., Krukenberg, K.A., and Mitchison, T.J. (2012). Large-scale preparation and characterization of poly(ADP-ribose) and defined length polymers. Anal. Biochem. *428*, 126–136.

Taylor, R.M., Thistlethwaite, A., and Caldecott, K.W. (2002). Central role for the XRCC1 BRCT I domain in mammalian DNA single-strand break repair. Mol. Cell. Biol. *22*, 2556–2563.

Wardlaw, C.P., Carr, A.M., and Oliver, A.W. (2014). TopBP1: A BRCT-scaffold protein functioning in multiple cellular pathways. DNA Repair (Amst.) *22*, 165–174.

Wei, L., Nakajima, S., Hsieh, C.L., Kanno, S., Masutani, M., Levine, A.S., Yasui, A., and Lan, L. (2013). Damage response of XRCC1 at sites of DNA single strand breaks is regulated by phosphorylation and ubiquitylation after degradation of poly(ADP-ribose). J. Cell Sci. *126*, 4414–4423.

Wielckens, K., Bredehorst, R., Adamietz, P., and Hilz, H. (1981). Protein-bound polymeric and monomeric ADP-ribose residues in hepatic tissues. Comparative analyses using a new procedure for the quantification of poly(ADP-ribose). Eur. J. Biochem. *117*, 69–74.

Williams, R.S., Lee, M.S., Hau, D.D., and Glover, J.N. (2004). Structural basis of phosphopeptide recognition by the BRCT domain of BRCA1. Nat. Struct. Mol. Biol. *11*, 519–525.

Wu, J., Liu, J., Zhou, Y., Ying, J., Zou, H., Guo, S., Wang, L., Zhao, N., Hu, J., Lu, D., Jin, L., Li, Q., and Wang, J.C. (2012). Predictive value of XRCC1 gene polymorphisms on platinum-based chemotherapy in advanced non-small cell lung cancer patients: a systematic review and meta-analysis. Clin. Cancer Res. *18*, 3972–3981.

Yuan, P., Liu, L., Wu, C., Zhong, R., Yu, D., Wu, J., Xu, Y., Nie, S., Miao, X., Sun, Y., et al. (2010). No association between XRCC1 polymorphisms and survival in non-small-cell lung cancer patients treated with platinum-based chemotherapy. Cancer Biol. Ther. *10*, 854–859.

Zeng, X.Y., Huang, J.M., Xu, J.W., Xu, Y., Yu, H.P., Ji, L., and Qiu, X.Q. (2013). Meta-analysis demonstrates lack of a relationship between XRCC1-399 gene polymorphisms and susceptibility to hepatocellular carcinoma. Genet. Mol. Res. *12*, 1916–1923.

STAR★METHODS

KEY RESOURCES TABLE

REAGENT or RESOURCE	SOURCE	IDENTIFIER
Antibodies		
His-affinity tag, mouse monoclonal	Takara Bio	Cat#631212
XRCC1 Antibody, rabbit polyclonal	Bio-techne	Cat#NBP1-87154
HRP-conjugated mouse anti-mouse IgG	GE healthcare	Cat#NA931
Bacterial and Virus Strains		
NEB 5-alpha Competent *E. coli*	New England Biolabs	Cat#C2987H
BL21(DE3) Competent *E. coli*	New England Biolabs	Cat#C2527I
Chemicals, Peptides, and Recombinant Proteins		
$[^{15}N]$ NH_4Cl	CortecNet	Cat#CN80P10
$[^{13}C3]$ glycerol	CortecNet	Cat#CC1065P10
Isopropyl β-D-1-thiogalactopyranoside	Generon	Cat#Gen-S-02122
HEPES	Fisher Scientific	Cat#10081113
NaCl	Fisher Scientific	Cat#10735921
Imidazole	Acros Organics	Cat#301870010
TCEP	Sigma-Aldrich	Cat#646547
cOmplete, EDTA-free Protease Inhibitor Cocktail	Sigma-Aldrich	Cat#4693159001
Talon resin	TaKaRa Bio	Cat#635503
Calf thymus histones	Sigma-Aldrich	Cat#H9250
Tris-HCl	Fisher Scientific	Cat#10316893
$MgCl_2$	Fisher Scientific	Cat#10386743
NAD+	Sigma-Aldrich	Cat#N8410
trichloroacetic acid	Fisher Scientific	Cat#10775151
EDTA	Fisher Scientific	Cat#10716481
KOH	Fisher Scientific	Cat#10448990
Ammonium acetate	Fisher Scientific	Cat#10365260
Guanidine HCl	Acros Organics	Cat#120230025
3-Aminophenylboronic acid monohydrate	Sigma-Aldrich	Cat#287512
Bio-Rex 70 Cation Exchange Resin	Bio-Rad	Cat#1425822
Urea	Fisher Scientific	Cat#10578260
Bromophenol blue	Fisher Scientific	Cat#10679733
Xylene cyanol	Sigma-Aldrich	Cat#X4126
Pierce Color Silver Stain Kit	Fisher Scientific	Cat#10096113
D_2O	Sigma-Aldrich	Cat#151882
6% DNA Retardation Gel	Fisher Scientific	Cat#12080086
Tris-borate-EDTA	Fisher Scientific	Cat#10542985
Gibson Assembly	New England Biolabs	Cat#E2611L
human PARP1	Trevigen	Cat#4668-02K-01
Tween 20	Sigma-Aldrich	Cat#P9416
3,3′,5,5′-tetramethylbenzidine	Sigma-Aldrich	Cat#T4319
Triton X-100	Sigma-Aldrich	Cat#T9284
Dulbecco’s Phosphate-Buffered Saline (PBS)	ThermoFisher Scientific	Cat#14190136
Hoechst 34580	Sigma-Aldrich	Cat#63493
Dulbecco’s modified Eagle’s medium (DMEM)	ThermoFisher Scientific	Cat#21969035
Foetal bovine serum	Sigma-Aldrich	Cat#F7524

(*Continued on next page*)

Continued

REAGENT or RESOURCE	SOURCE	IDENTIFIER
Glutamine	ThermoFisher Scientific	Cat#25030081
Penicillin-Streptomycin	Sigma-Aldrich	Cat#P4333
Genejuice	Novagen	Cat#70967
Blasticidin	InvivoGen	Cat#ant-bl-1
Methyl methanesulfonate	Sigma-Aldrich	Cat#129925
Low-gelling-temperature agarose, Type VII-A	Sigma-Aldrich	Cat#A0701
DMSO	Sigma-Aldrich	Cat#276855
SybrGreen I	Fisher Scientific	Cat#S7563
Hydrogen peroxide	Sigma-Aldrich	Cat#H1009
Paraformaldehyde	Agar Scientific	Cat#AGR1026
Hoechst 33342	Sigma-Aldrich	Cat#B2261
ECL reagent	Fisher Scientific	Cat#10455145
PD MidiTrap G-10 column	Sigma-Aldrich	Cat#GE28-9180
Tankyrase 1	(Elliott et al., 2015)	N/A
Deposited Data		
Biological Magnetic Resonance Bank		27598
Experimental Models: Cell Lines		
Osteosarcoma U2-OS	Genome Damage and Stability Centre cell repository	ID: U2-OS
Oligonucleotides		
Oligonucleotides for DNA-binding experiments, see Figure S1.	Integrated DNA Technologies	N/A
Recombinant DNA		
pET15b	Novagen	69661
peGFP-N1	Clontech	6085-1
pET15b-SUMO-XRCC1-BRCT1	This paper	N/A
Software and Algorithms		
GraphPad Prism7 for Mac OS X	Graphpad	https://www.graphpad.com/scientific-software/prism/
SlideBook 6	3i	https://www.intelligent-imaging.com/slidebook
CcpNmr Analysis	Collaborative Computing Project for NMR	https://www.ccpn.ac.uk/
Comet Assay IV software	Perceptive Instruments	http://www.scorecomets.com/comet-scoring/comet-assay-iv
Harmony high-content analysis software	PerkinElmer	Cat#HH17000001
ImageJ64	ImageJ Software	https://imagej.nih.gov/ij/
Other		
BD FACSMelody	BD Biosciences	N/A
Operetta CLS high-content analysis system	PerkinElmer	Cat#HH16000000
3i Spinning Disk Confocal microscope	3i	N/A

CONTACT FOR REAGENT AND RESOURCE SHARING

Further information and requests for resources and reagents should be directed to and will be fulfilled by the Lead Contact, Prof. Laurence Pearl FRS (Laurence.Pearl@sussex.ac.uk).

EXPERIMENTAL MODEL AND SUBJECT DETAILS

Cell culture

The osteosarcoma cell line U2-OS (obtained from the Genome Damage and Stability Centre cell repository) was maintained as monolayers in Dulbecco's modified Eagle's medium (DMEM), supplemented with 10% (vol/vol) fetal bovine serum, 100 U/ml penicillin, 2 mM glutamine and 100 μg/ml streptomycin.

METHOD DETAILS

Cloning

DNA encoding the required region of human XRCC1 was amplified by PCR from human cDNA. DNA encoding human 6xHis-SUMO-XRCC1-BRCT1$^{301\text{-}410}$ was amplified by PCR, using synthetic DNA codon-optimized for expression in *E. coli* as a template (Genscript, Piscataway, USA). Primers were designed to sub-clone the amplified DNA into vectors suitable for protein expression in *E. coli* by Gibson Assembly (New England Biolabs).

Expression and purification

E. coli strain BL21(DE3) (Merck Millipore) was co-transformed with pET15b-SUMO-XRCC1-BRCT1 plasmid. Transformants were selected on LB-agar plates added with antibiotics. From an overnight culture, 25 ml was used to inoculate a 2 l flask, containing 1 l of Turbo-broth media (Molecular Dimensions, Newmarket, UK) again supplemented with antibiotics. Cultures were grown in an orbital-shaking incubator, at 37°C, until an optical density of ~1.5 units at a wavelength of 600 nm was reached. The temperature was then reduced to 20°C, and recombinant protein expression induced by the addition of 0.15 M isopropyl β-D-1-thiogalactopyranoside. Cells were subsequently harvested by centrifugation after 16 h at the reduced temperature. The resultant pellet was stored at −20°C until required.

For NMR experiments, the protein was expressed in 1 l filter-sterilized Overnight Express Autoinduction NMR Media (Merck-Millipore, Billerica, MA, USA) containing 50 mM [^{15}N] NH4Cl and 0.5% (w/v) [^{13}C3] glycerol (CortecNet, Voisins-le-Bretonneux, France) at a temperature of 25°C for 30 h.

The cell pellet resulting from 4 l of culture was resuspended in Buffer A (50 mM HEPES.NaOH pH 7.5, 250 mM NaCl, 10 mM imidazole, 0.5 mM TCEP) supplemented with protease inhibitor tablets (Roche, Burgess Hill, UK). Cells were then disrupted by sonication, and insoluble material removed by centrifugation. The resultant supernatant was incubated with Talon resin (TaKaRa Bio) pre-equilibrated in Buffer A. After successive washes with Buffer A to remove unbound material, the retained recombinant proteins were eluted by the additon of Buffer B (50 mM HEPES.NaOH pH 7.5, 250 mM NaCl, 300 mM imidazole, 0.5 mM TCEP). The affinity tag and SUMO were then cleaved by overnight incubation with SENP1 at 4°C. The proteins were concentrated to a final volume of 3 ml using Vivaspin 20 (10,000 MWCO) centrifugal concentrators (Sartorius Stedim Biotech, Goettingen, Germany) and then loaded onto a Superdex 75 size exclusion chromatography column (GE Healthcare Life Sciences, Little Chalfont, UK) pre-equilibrated with Buffer C (20 mM HEPES.NaOH pH 7.5, 250 mM NaCl, 0.5 mM TCEP) as the final purification step. Fractions containing the purified complex were identified by SDS-PAGE, pooled and then concentrated to 11 mg ml^{-1} and either used immediately or flash-frozen in liquid N_2 and stored at −80°C until required.

Poly(ADP-ribose) preparation

The purification protocol is based on (Tan et al., 2012) with some minor alterations. The PARylation reaction was as follows: 1mg/ml calf thymus histones (Sigma-Aldrich) in PARP reaction buffer (50mM Tris-HCl pH 8, 0.8mM MgCl2, 1% v/v glycerol and 0.5mM DTT), 200mM NAD+ (Sigma-Aldrich) were mixed with 1mg/ml tankrase 1 enzyme. The reaction was stopped after 1 hour at room temperature by adding an equal volume of 20% v/v ice-cold trichloroacetic acid, and incubated on ice for 15 min. The precipitated ribosylated protein was pelleted by centrifugation at top speed at 4°C, dissolved in 100μl 1M KOH/50mM EDTA and was incubated for 60 min at 60°C. Then, AAGE9 buffer (250mM ammonium acetate, 6M guanidine HCl, 10mM EDTA, pH 9.0) was added and the sample was loaded onto 1ml dihydroxyboryl Bio-Rex resin pre-equilibrated with AAGE9 buffer. The dihydroxyboryl resin was prepared by coupling BioRex 70 beads, (100-200 mesh, Bio-Rad) and N-ethyl-N'-(3-diethylaminopropyl)-carbodiimide (Sigma-Aldrich), as described (Wielckens et al., 1981). The resin was washed with 10 column volumes (cv) AAGE9 buffer, 20cv 1M ammonium acetate pH 9.0 buffer, eluted with 6cv water and collected in 1cv fractions. Successive fractions were analyzed by UV spectroscopy (258nm), using a NanoDrop2000 (Thermo Fisher Scientific) and fractions containing bulk PAR were loaded onto a 1ml MonoQ 5/50 chromatography column (GE Healthcare). The column was extensively washed with Buffer A (25mM Tris-HCl, pH9.0) to remove any unbound material. Bound PAR was eluted by the application of the following linear gradient series from Buffer A to Buffer B (25mM Tris-HCl, pH 9.0, 1M NaCl): 0% to 15% B over 5cv, then 15% to 40% B over 130 cv, followed by 40% to 45% B over 80cv, and a final step from 45% to 100% B over 3cv. Fractions were dried in a Savant DNA120 SpeedVacTM concentrator (Thermo Fisher Scientific) and stored at −20°C until required. Fractions containing PAR with the same elution volume, were loaded together onto a PD MidiTrap G-10 column (GE Healthcare) pre-equilibrated with water, eluted following the manufacturer's protocol and again dried before being stored at −20°C. Purified PAR fractions were adjusted to a final concentration of 0.3μM and then diluted in loading buffer (40% w/v urea, 4mM EDTA, 0.02% w/v Bromophenol blue, and 0.02% w/v Xylene cyanol) to a final volume of 15μl and then loaded onto a 20% v/v

polyacrylamide gel (Thermo Fisher Scientific) containing 1x TBE buffer. The gel was run at a constant power of 15W until the dye front migrated approximately 50% of the gel; after which the gel was fixed in a 50% v/v ethanol and 5% v/v acetic acid solution for 2 hours, and washed with ultrapure water. It was stained with a Pierce Color Silver Stain Kit (Thermo Fisher Scientific) following manufacturer's protocol.

NMR resonance assignment

NMR spectra were recorded at 303K on Bruker DRX600 and DRX800 spectrometers equipped with cryo-probes. XRCC1-BRCT1 was dissolved in 300 μl NMR buffer containing 20 mM Tris-HCl, pH 7.5, 125 mM NaCl, 1 mM TCEP and 10% D_2O to a final concentration of ~350 μM. The chemical shifts of ^{1}HN, ^{15}N, ^{13}Cα, ^{13}Cβ and ^{13}CO cross-peaks were assigned using CBCA(CO)NH, HNCACB, HNCO and HN(CA)CO experiments and data were analyzed using the program CCPNMR Analysis (Skinner et al., 2016). > 90% of the amino acid backbone resonances were assigned. A similar procedure was followed to assign chemical shifts after formation of complexes between XRCC1-BRCT1 and DNA (oligonucleotides detailed in Figure S1) or Poly (ADP-ribose). For binding of DNA to the BRCT1-PAR complex, the BRCT1 was saturated by addition of PAR4 until no further chemical shift was obtained. After saturation with PAR4 and DNA, peaks were reassigned in the HSQC spectrum using HNCA data. Chemical shift perturbations (CSP) were calculated as: $[^1H\Delta^2 + (0.15{*}^{15}N\Delta)\ ^2]^{0.5}$. NMR data has been deposited in the Biological Magnetic Resonance Bank with accession number 27598.

Electrophoretic Mobility Shift Assay

Oligonucleotides at a concentration of 100 nM, were mixed with increasing concentrations of constructs of XRCC1, in 20 mM HEPES.NaOH pH 7.5, 100 mM NaCl, 1 mM EDTA, 0.5 mM TCEP, and incubated for 10 min at room temperature. Samples were then analyzed on 6% v/v native polyacrylamide gels (6% DNA Retardation Gel, ThermoFisher Scientific) containing 0.5X tris-borate-EDTA (TBE) and visualized by direct scanning of the gel in a Fuji FLA-5100 Fluorescent Image Analyzer.

Fluorescence polarization

Fluorescent dsDNA oligonucleotides were assembled as shown in Figure S1, with fluorescein isothiocyanate attached to the 5′-terminus of the continuous strand. For fluorescent polarization experiments, annealed oligonucleotides at a concentration of 10 μM were incubated with increasing concentrations of wild-type XRCC1-BRCT1, in 20 mM HEPES.NaOH pH7.5, 100 mM NaCl, 1 mM EDTA, 0.5 mM TCEP, and incubated for 10 min at room temperature. Fluorescence polarization was measured in a POLARstar OMEGA multimode plate reader (BMG Labtech GmbH, Offenburg, Germany).

Poly (ADP-ribose) binding assays

The wells of flat bottomed 96 well PS-microplates (Greiner) were incubated with either 50 μL recombinant histone H1 at 0.1 mg/ml in phosphate buffered saline (PBS) overnight at 4°C and the wells rinsed (4 ×) with 0.2 mL 0.1% Triton X-100 in PBS. The adsorbed proteins were mock ribosylated in the absence of NAD+ or ribosylated in the presence of the 50 mM NAD+ (Sigma) in PARP1 reaction buffer (50 mM Tris–HCl pH7.5, 0.8 mM $MgCl_2$, 1% glycerol and 1.5 mM DTT) containing 40 nM single-stranded oligodeoxyribonucleotide (5′-CATATGCCGGAGATCCGCCTCC-3′) and 5 nM human PARP1 (Trevigen) in a final volume of 50 μL at room temp for 30 min. After rinsing (4 ×) with 50 μL of 0.1% Tween 20 in PBS, 50 μL of His-SUMO-XRCC-BRCT1 or its variants (diluted to 25 nM in 20 mM Tris pH7.5, 130 nM NaCl) were added to the adsorbed proteins and incubated on ice for 30 min. The wells were then rinsed (4 ×) as above and incubated with 50 μL mouse anti-polyhistidine (His-tag) Mab (Takara Bio, diluted 1:2500 in 20 mM Tris pH7.5, 130 nM NaCl) followed by 50 μL HRP-conjugated mouse anti-mouse IgG (ECL, GE Healthcare, 1: 5000 in dilution buffer) for 30 min each on ice. After a final wash with 3,3′,5,5′-tetramethylbenzidine liquid substrate, slow kinetic form (Sigma-Aldrich) was added to the wells, incubated in the dark for 10 min, stopped by adding 0.2 M HCl, and the absorbance was read at 450 nm.

UVA-laser micro-irradiation

ORFs encoding human XRCC1-R399-GFP was generated by PCR amplification of the human XRCC1 ORF and subcloning using Gibson Assembly (New England Biolabs) in peGFP-N1. Point mutations in the BRCT1 domain were generated by site-directed mutagenesis. Osteosarcoma U2-OS cells were seeded onto glass-bottom dishes (Nunc, Thermo Scientific) and transfected with 1 μg of the indicated GFP constructs 24 h before micro-irradiation and incubated with 10 μg ml^{-1} Hoechst 34580 for 30 min before irradiation. Cells were micro-irradiated with a 405 nm UV-laser at a dose of 0.22 μJ μm^{-2} (Breslin et al., 2015), and time-lapse images recorded at 0.5 s intervals for a total of 3 min per cell using a Spinning Disk Confocal microscope (3i).

Generation of gene-edited U2-OS cells

XRCC1 gene edited U2OS cells, denoted in the figures as *XRCC1*$^{-/-}$ for simplicity, were generated using the Cas9 and XRCC1 guide expression constructs as previously described (Hanzlikova et al., 2017). This cell line will be described in detail, elsewhere. Successful gene editing was confirmed by Sanger sequencing and by western blotting (Figure S2).

Cell lines expressing XRCC1

The cell lines with U2OS-GFP-XRCC1 WT and its variants were generated by transfection of 1 × 10^6 *XRCC1*$^{-/-}$ U2OS cells with 0.5 μg of vectors by Genejuice transfection (Novagen). Twenty four hours after transfection, cells were selected in media containing 7.5 μg ml^{-1} of Blasticidin (InvivoGen) for 3 weeks, and a population of cells were selected based on their level of GFP expression by using a Melody cell sorter (BD).

Alkaline comet assays

Osteosarcoma U2-OS cells were treated with 0.1 and 0.2 mg/ml of MMS at 37°C. Cells were then suspended in pre-chilled Dulbecco's PBS and mixed with an equal volume of 1.2% low-gelling-temperature agarose (Sigma, type VII) maintained at 42°C. Cell suspension was immediately layered onto pre-chilled frosted glass slides (Fisher) pre-coated with 0.6% agarose and maintained in the dark at 4°C until set, and for all further steps. Slides were immersed in pre-chilled lysis buffer (2.5 M NaCl, 10 mM Tris-HCl, 100 mM EDTA, 1% Triton X-100, 1% DMSO; pH10) for 1 h, washed with pre-chilled distilled water (2 × 5 min), and placed for 45 min in pre-chilled alkaline electrophoresis buffer (50 mM NaOH, 1 mM EDTA, 1% DMSO). Electrophoresis was then conducted at 1 V/cm for 25 min, followed by neutralization in 400 mM Tris-HCl pH7.4 overnight. Finally, DNA was stained with SybrGreen I (1:10,000 in PBS) for 30 min. Average tail moments from 100 cells/sample were measured using Comet Assay IV software (Perceptive Instruments, UK). Data are the average ± 1 SEM of three independent experiments and were scored blind.

Chromatin associated XRCC1

XRCC1$^{-/-}$-U2OS stably expressing GFP-XRCC1 WT and variants were mock-treated or treated with 1 mM hydrogen peroxide (H_2O_2) for 10 min, incubated at 37°C in drug free media for indicated times, pre-extracted with 0.2% Triton X-100 for 2 min, washed with phosphate buffered saline (PBS), then fixed for 10 min in 4% paraformaldehyde in PBS at room temperature and stained with Hoechst 33342 (blue, Sigma-Aldrich, B2261).

Cell survival assay

Clonogenic survival was determined by colony formation assays. Briefly, U2-OS cells were counted and plated in DMEM medium containing 10% FBS. Cells were treated with MMS and after incubation for 10 days colonies that were visible by eye were counted. Survival was calculated by dividing the number of colonies in treated wells by those in untreated wells.

SDS-PAGE and western blotting

Cells were collected and lysed in SDS sample buffer (2% SDS, 10% glycerol, 50 mM Tric-Cl, pH 6.8), denatured for 10 min at 95°C, and sonicated for 30 s using Bioruptor® Pico (Diagenode). Samples were subjected to SDS-PAGE, proteins transferred onto nitrocellulose membrane and detected by relevant specific antibodies combined with horseradish peroxidase-conjugated secondary antibodies. Peroxidase activity was detected by ECL reagent (GE Healthcare) in an ImageQuant LAS-4000 image reader (GE life sciences). Primary antibodies: His-affinity tag, mouse monoclonal at 1:5000 dilution (631212, Takara Bio) and XRCC1 Antibody, rabbit polyclonal at 1:2000 (NBP1-87154, Bio-techne). Band intensities were determined using ImageJ64 (ImageJ Software).

QUANTIFICATION AND STATISTICAL ANALYSIS

Statistical analyses were performed using GraphPad Prism. Binding data from fluorescent polarization were analyzed with GraphPad Prism 7.0, by non-linear fitting with a one-site binding model, to give the reported dissociation constants (Kd). All data from fluorescent polarization experiments represent the mean of four measurements comprised of two separate replicates with XRCC1-BRCT1 from two separate protein purifications. Data from Poly (ADP-ribose) binding assays was the result of represent the mean of four measurements of three separate replicates and it was analyzed by two-way ANOVA. Average comet tail moments from 100 cells/sample were measured using Comet Assay IV software (Perceptive Instruments, UK). Quantification of detergent-insoluble XRCC1 signal from > 8000 cells per sample per experiment using Perkin-Elmer Operetta analysis software and analyzed by two-way ANOVA. Comet assay data and cell survival results were scored blind and are shown as the average ± 1 SEM of three independent experiments, compared using two-way ANOVA. Western blot band intensities were analyzed by one-way ANOVA.

Chemical shift perturbations (CSP) were calculated as: $[{}^{1}H\Delta^{2} + (0.15*{}^{15}N\Delta)^{2}]^{0.5}$.

DATA AND SOFTWARE AVAILABILITY

NMR assignment data have been deposited into the Biological Magnetic Resonance Data Bank with accession number 27598. No other large datasets are associated with this work. All other data are available from the authors on request.

Cell Reports, Volume *26*

Supplemental Information

Efficient Single-Strand Break Repair Requires Binding to Both Poly(ADP-Ribose) and DNA by the Central BRCT Domain of XRCC1

Luis M. Polo, Yingqi Xu, Peter Hornyak, Fernando Garces, Zhihong Zeng, Richard Hailstone, Steve J. Matthews, Keith W. Caldecott, Antony W. Oliver, and Laurence H. Pearl

FIGURE S1 - OLIGONUCLEOTIDE STRUCTURES AND AFFINITIES - related to FIGURE 1

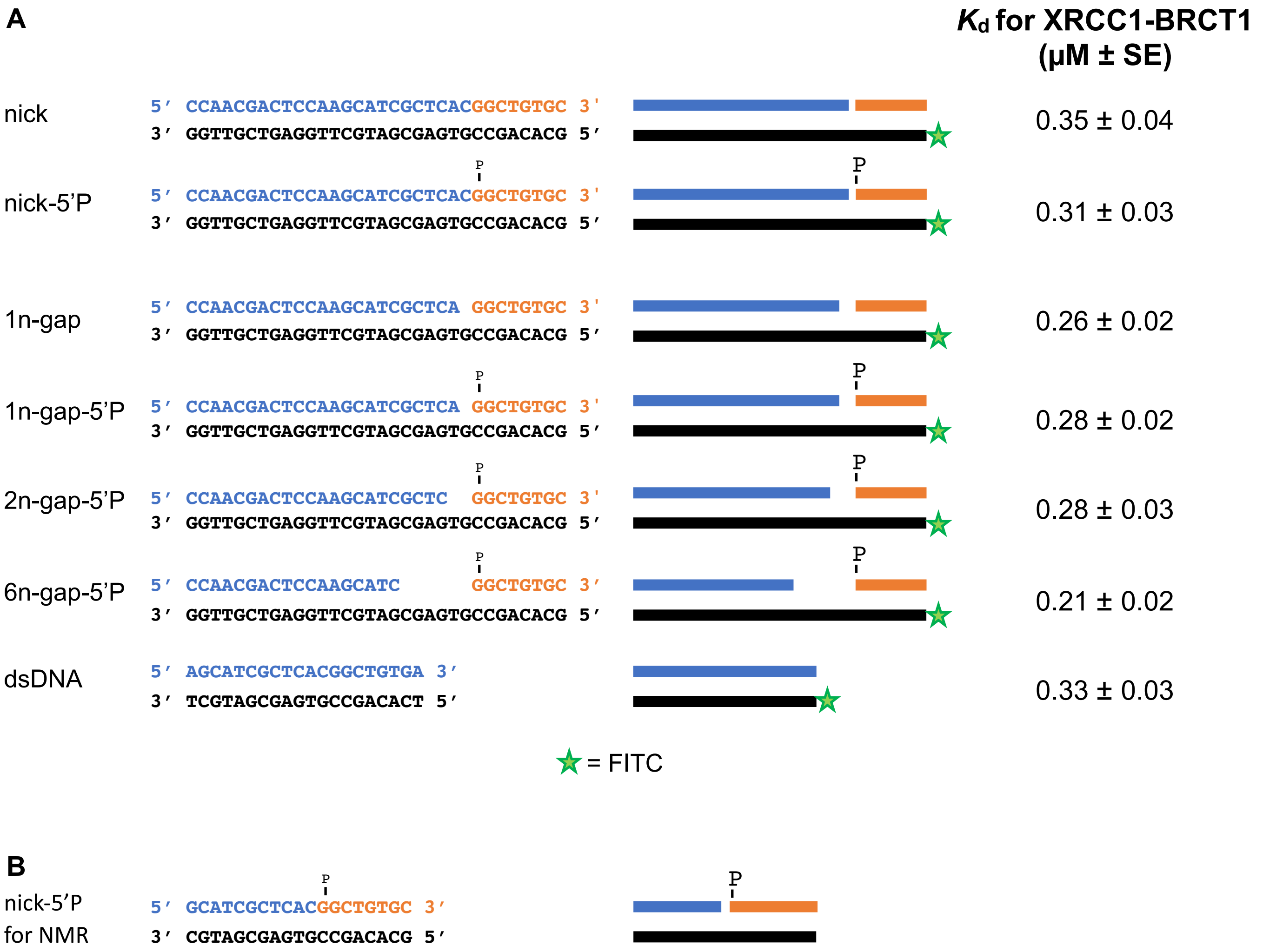

A. Schematics of DNA oligonucleotide duplexes used in this study, and their Kd for binding to XRCC1-BRCT1. The positions of 5'-phosphorylations are indicated by 'P' and the green star indicates the addition of the fluorescent dye fluorescein isothiocyanate (FITC).

B. Schematic of DNA oligonucleotide duplex used in NMR studies.

FIGURE S2 - Chemical Shift Perturbations - related to FIGURE 2

BRCT1
+ PAR4

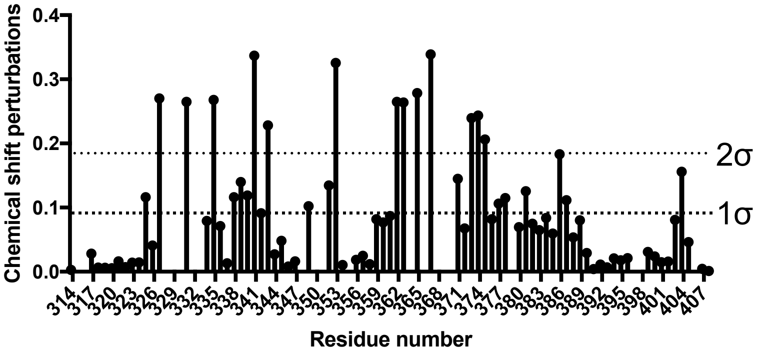

BRCT1
+ DNA

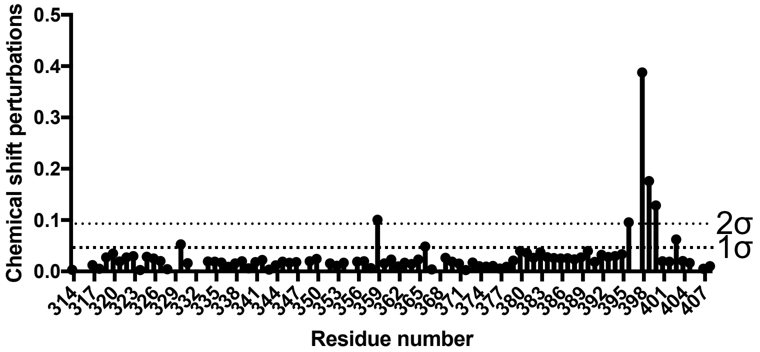

BRCT1-PAR4
+ DNA

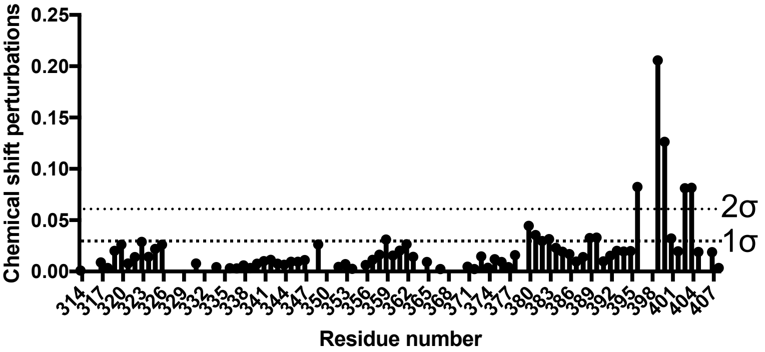

Chemical shift perturbations (CSP) by residue - residues whose CSP exceeds 2 standard deviations from the mean CSP for the experiment are highlighted in Figure 2e,f

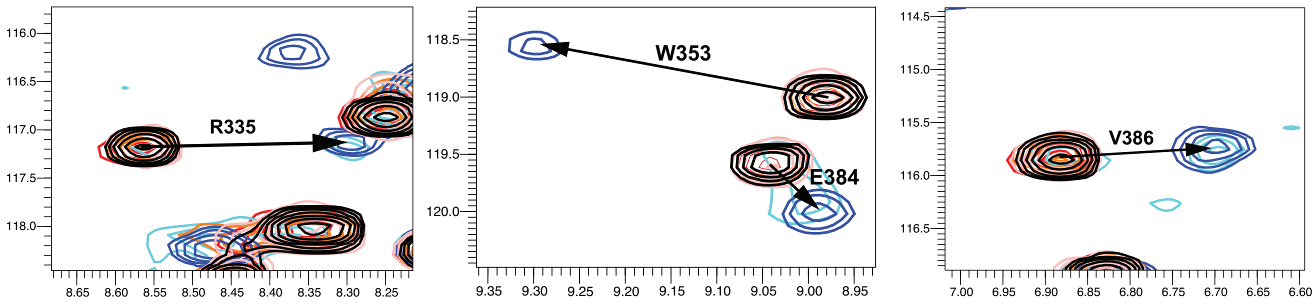

Protein:PAR	apo	1:1	1:2	1:4	1:10
Colour	black	pink	orange	cyan	blue

Example chemical shift perturbations (CSP) as a function of protein:PAR4 ratio. All shifts are fully saturated at 1:10 ratio.

FIGURE S3 - OLIGONUCLEOTIDE-BINDING AFFINITIES OF XRCC1-BRCT1 MUTANTS - related to FIGURE 3

	K_d for XRCC1-BRCT1 (μM ± SE)	
	oligonucleotide	
**XRCC1-BRCT1 Variant/Mutant**	**nick**	**nick 5’Pi**
R399	0.31 ± 0.04	0.32 ± 0.04
Q399	0.42 ± 0.1	1.45 ± 0.26
R399D/R400Q	not determined	not determined
R335A/K369A	0.33 ± 0.09	1.16 ± 0.32

Binding affinities of oligonucleotides (see FIGURE S1A) for different XRCC1-BRCT1 mutants/variants.

FIGURE S4 - XRCC1 EXPRESSION LEVELS AND COMET ASSAYS - related to FIGURE 4

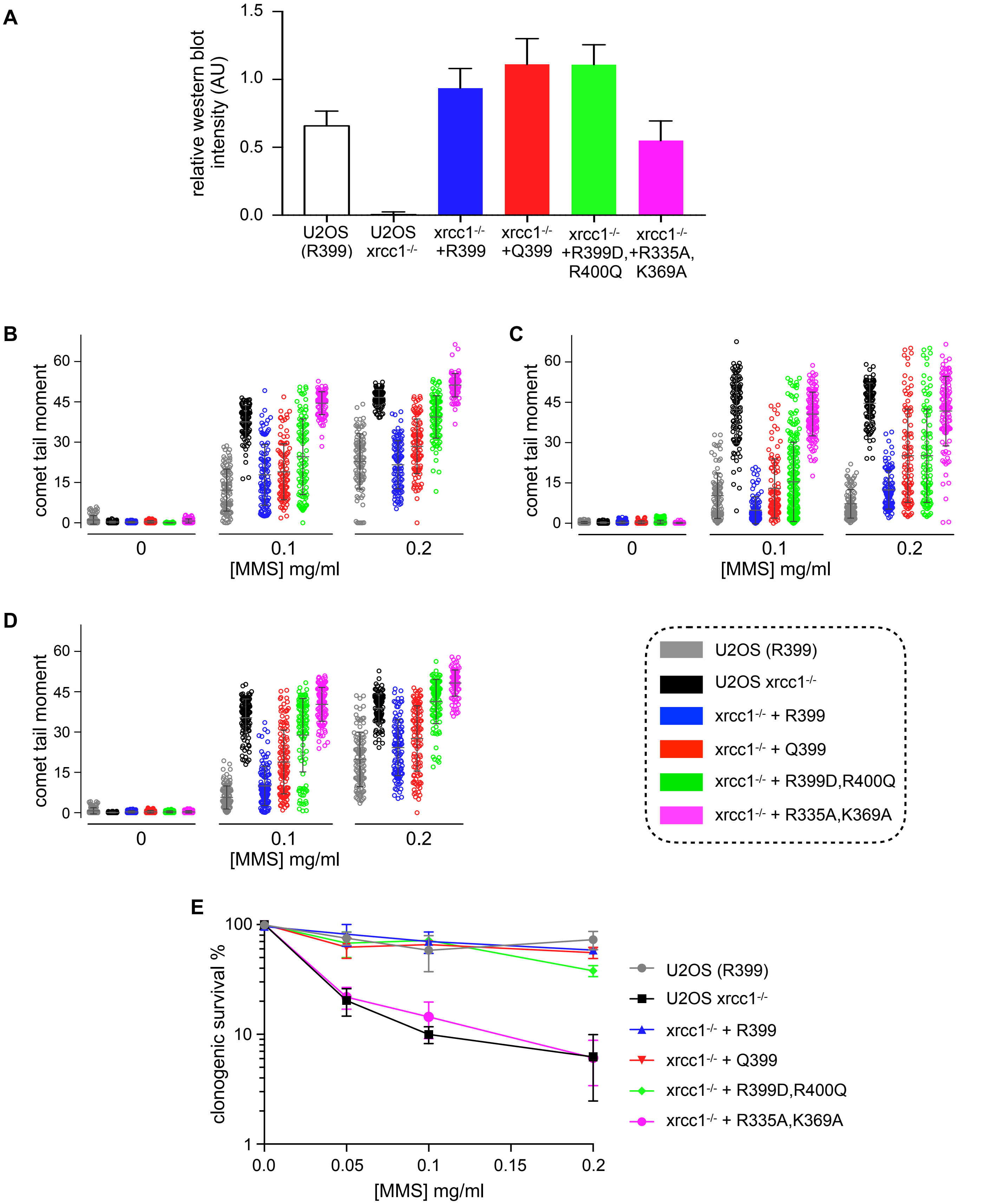

A. Relative expression levels of XRCC1 proteins determined by western blot intensity, in : U2OS cells, *XRCC1* gene edited cells, and gene edited cells with different XRCC1 variants or mutants added back. All mutants and variants are expressed at least at levels comparable to the wild-type U2OS cells.

B,C,D Replicate alkaline comet assays summarised in **FIGURE 4C.** The legend is shown in the dashed cartouche.

E. Clonogenic survival assay for U2OS variants and mutants (see **METHODS**).
